# Yeast strains do have an impact on the production of cured cocoa beans, as assessed with Costa Rican Trinitario cocoa fermentation processes and chocolates thereof

**DOI:** 10.3389/fmicb.2023.1232323

**Published:** 2023-08-09

**Authors:** Dario Van de Voorde, Cristian Díaz-Muñoz, Carlos Eduardo Hernandez, Stefan Weckx, Luc De Vuyst

**Affiliations:** ^1^Research Group of Industrial Microbiology and Food Biotechnology, Faculty of Sciences and Bioengineering Sciences, Vrije Universiteit Brussel, Brussels, Belgium; ^2^Laboratorio de Calidad e Innovación Agroalimentaria, Escuela de Ciencias Agrarias, Universidad Nacional de Costa Rica, Heredia, Costa Rica

**Keywords:** cocoa fermentation, cured cocoa, starter cultures, yeasts, lactic acid bacteria, acetic acid bacteria, chocolate-making

## Abstract

The microbiological and metabolic outcomes of good cocoa fermentation practices can be standardized and influenced through the addition of starter culture mixtures composed of yeast and bacterial strains. The present study performed two spontaneous and 10 starter culture-initiated (SCI) cocoa fermentation processes (CFPs) in Costa Rica with local Trinitario cocoa. The yeast strains *Saccharomyces cerevisiae* IMDO 050523, *Hanseniaspora opuntiae* IMDO 020003, and *Pichia kudriavzevii* IMDO 060005 were used to compose starter culture mixtures in combination with the lactic acid bacterium strain *Limosilactobacillus fermentum* IMDO 0611222 and the acetic acid bacterium strain *Acetobacter pasteurianus* IMDO 0506386. The microbial community and metabolite dynamics of the cocoa pulp-bean mass fermentation, the metabolite dynamics of the drying cocoa beans, and the volatile organic compound (VOC) profiles of the chocolate production were assessed. An amplicon sequence variant approach based on full-length 16S rRNA gene sequencing instead of targeting the V4 region led to a highly accurate monitoring of the starter culture strains added, in particular the *Liml. fermentum* IMDO 0611222 strain. The latter strain always prevailed over the background lactic acid bacteria. A similar approach, based on the internal transcribed spacer (ITS1) region of the fungal rRNA transcribed unit, was used for yeast strain monitoring. The SCI CFPs evolved faster when compared to the spontaneous ones. Moreover, the yeast strains applied did have an impact. The presence of *S. cerevisiae* IMDO 050523 was necessary for successful fermentation of the cocoa pulp-bean mass, which was characterized by the production of higher alcohols and esters. In contrast, the inoculation of *H. opuntiae* IMDO 020003 as the sole yeast strain led to underfermentation and a poor VOC profile, mainly due to its low competitiveness. The *P. kudriavzevii* IMDO 060005 strain tested in the present study did not contribute to a richer VOC profile. Although differences in VOCs could be revealed in the cocoa liquors, no significant effect on the final chocolates could be obtained, mainly due to a great impact of cocoa liquor processing during chocolate-making. Hence, optimization of the starter culture mixture and cocoa liquor processing seem to be of pivotal importance.

## 1. Introduction

Cured cocoa beans are the principal raw material for the production of cocoa-related products, such as chocolate (Afoakwa et al., 2008; De Vuyst and Leroy, 2020; Santander Muñoz et al., 2020). Curing involves fermentation and drying. These post-harvest processing steps of the cocoa pulp-bean mass, derived from the cocoa fruits of the cocoa tree (*Theobroma cacao* L.), are decisive for the flavour potential of the cured cocoa beans, as both flavour precursors that are important for further cocoa processing and flavour-active molecules are then formed (Kongor et al., 2016; De Vuyst and Leroy, 2020). The microbial succession during spontaneous cocoa fermentation processes (CFPs) is mainly determined by yeasts, lactic acid bacteria (LAB), and acetic acid bacteria (AAB); their different growth extent and metabolic activities result in complex flavour profiles of both cured cocoa beans and chocolates produced thereof (Schwan and Wheals, 2004; Lima et al., 2011; De Vuyst and Weckx, 2016; Figueroa-Hernández et al., 2019; Santander Muñoz et al., 2020; De Vuyst and Leroy, 2020). In the past, several starter culture-initiated (SCI) CFPs dealt with solely yeast species (Sanchez et al., 1985; Cempaka et al., 2014; Ramos et al., 2014; Meersman et al., 2015; Batista et al., 2016; Menezes et al., 2016), LAB species (Kresnowati et al., 2013), or AAB species (Samah et al., 1993). Other SCI CFPs dealt with starter culture mixtures that combined yeast, LAB, and/or AAB species (Schwan, 1998; Lefeber et al., 2012; Crafack et al., 2013, 2014; Sandhya et al., 2016; Miguel et al., 2017; Moreira et al., 2017; Díaz-Muñoz et al., 2021, 2023). Indeed, knowledge of the most prevalent yeast, LAB, and AAB species and their metabolism has seeded intensive research on candidate starter culture strains and the formulation of mixed-strain starter cultures to initiate CFPs (Lefeber et al., 2010, 2011a,b, 2012; Illeghems et al., 2012, 2013, 2015a,b; Papalexandratou et al., 2013; Batista et al., 2015; Apriyanto, 2017; Visintin et al., 2017; De Vuyst and Leroy, 2020). Consequently, the increasing economic importance of cocoa products and an improved fermentation outcome by applying starter cultures has increased the relevance of SCI cocoa fermentation trials (Saltini et al., 2013; De Vuyst and Weckx, 2016; Pereira et al., 2016; Ozturk and Young, 2017; Castro-Alayo et al., 2019; De Vuyst and Leroy, 2020; Díaz-Muñoz et al., 2021, 2023; Lima et al., 2021).

The yeast species diversity of the fermenting cocoa pulp-bean mass is rather broad compared to that of the LAB and AAB species (Jespersen et al., 2005; Daniel et al., 2009; Papalexandratou and De Vuyst, 2011; Maura et al., 2016; De Vuyst and Leroy, 2020; Díaz-Muñoz et al., 2021, 2023). Previous spontaneous and SCI CFPs regarding yeasts encompassed strains of species of *Hanseniaspora*, *Pichia*, and *Saccharomyces* and have shown that these species are key participants among the yeast communities (Daniel et al., 2009; Papalexandratou and De Vuyst, 2011; Meersman et al., 2013; Papalexandratou et al., 2013; De Vuyst and Weckx, 2016; Maura et al., 2016; De Vuyst and Leroy, 2020; Díaz-Muñoz et al., 2021, 2023). Moreover, the cruciality of strains of LAB and AAB species for successful CFPs has been a subject of debate, although their contributions are relevant (Ho et al., 2015, 2018; Meersman et al., 2015; Moreira et al., 2017; De Vuyst and Leroy, 2020; John et al., 2020). However, given the high inter-and even intraspecies diversity of the yeast communities, yeasts are supposed to have a greater impact on the fermentation extent of the cocoa pulp-bean mass and to influence the cured cocoa bean quality substantially. Indeed, many volatile organic compounds (VOCs) and non-VOCs of flavour impact are produced via the yeast metabolism during fermentation of the cocoa pulp-bean mass, such as higher aldehydes, higher alcohols, and esters (Schwan and Wheals, 2004; Aculey et al., 2010; Rodriguez-Campos et al., 2011, 2012; Ho et al., 2014; Ramos et al., 2014; Koné et al., 2016; Menezes et al., 2016; De Vuyst and Leroy, 2020; Díaz-Muñoz et al., 2021, 2023).

As *Hanseniaspora* species, such as *Hanseniaspora opuntiae*, possess a low ethanol and heat tolerance, have a good acid tolerance, and appear to be non-competitive with citrate-converting fructose-loving LAB species (Daniel et al., 2009; Papalexandratou and De Vuyst, 2011; Hamdouche et al., 2015; De Vuyst and Leroy, 2020; Díaz-Muñoz et al., 2023), they frequently occur during the initial phase of CFPs, under low-ethanol and low-pH conditions and in the presence of citrate. They seldomly prevail during the whole fermentation course (Daniel et al., 2009; Papalexandratou et al., 2013; Díaz-Muñoz et al., 2021), as they have to compete for substrates with more ethanol-tolerant yeast species, such as *Saccharomyces cerevisiae* and *Pichia* species. Among the latter, *Pichia kudriavzevii* often prevails toward the end of CFPs, either solely or together with *S. cerevisiae* (Papalexandratou et al., 2011b,c, 2013; Díaz-Muñoz et al., 2021). Both yeast species are the main contributors to flavour formation upon fermentation (Jespersen et al., 2005; Daniel et al., 2009; Meersman et al., 2013; Pereira et al., 2017; Díaz-Muñoz et al., 2021). For instance, the application of a mixed-strain starter culture composed of *S. cerevisiae* IMDO 050523 and *P. kudriavzevii* IMDO 020508 leads the VOC production toward isoamyl acetate or 3-methyl butanal, 2-phenyl ethanol and ethyl decanoate, respectively (Díaz-Muñoz et al., 2021). However, the production of flavour-active compounds by yeast species is often strain-dependent (Meersman et al., 2015, 2016; Papalexandratou and Nielsen, 2016; Pereira et al., 2017; Assi-Clair et al., 2019). Hence, it is not clear to what extent all these yeast species do contribute, either solely or in combination, to the flavour quality of cured cocoa beans and chocolates produced thereof. The investigation of several potential starter culture mixtures, including strains of *Hanseniaspora* or *Pichia* species, may help to elucidate this (Pereira et al., 2012, 2017; Ho et al., 2018; Ooi et al., 2020, 2021; Díaz-Muñoz et al., 2021, 2023). Therefore, different strains of both yeast species should be tested in a systematic way, applying both whole-community analysis and fine-scale monitoring of the strains inoculated in the cocoa pulp-bean mass as well as a temporal follow-up of the substrates degraded and metabolites produced, including VOCs, the latter not only in the cocoa pulp and beans but also during cocoa bean processing up to the final chocolates. Fine-scale monitoring has become possible by means of an amplicon sequence variant (ASV) approach (Callahan et al., 2019; Díaz-Muñoz et al., 2021, 2023). However, it is not always straightforward to assign the LAB and AAB ASVs to specific strains when only considering the V4 region of the 16S rRNA gene. Therefore, targeting the full-length 16S rRNA gene and the internal transcribed spacer (ITS1) region of the fungal rRNA transcribed unit will enable monitoring of the inoculated strains in a more accurate fashion (Callahan et al., 2019).

Finally, studies dealing with CFPs performed with the Trinitario cocoa variety are scarce (Cevallos-Cevallos et al., 2018; Ho et al., 2018; Castro-Alayo et al., 2019; Rottiers et al., 2019; Romanens et al., 2020; Verce et al., 2021; Alvarez-Villagomez et al., 2022), in particular when carried out in Costa Rica (Di Mattia et al., 2013; Mayorga-Gross et al., 2016; Díaz-Muñoz et al., 2021, 2023; Verce et al., 2021). Given the fine flavour quality of Trinitario cocoa, the impact of the use of starter cultures may have been underestimated compared to their use for the improvement of the fermentation of bulk cocoa varieties, such as Forastero (Rodriguez-Campos et al., 2011, 2012; Lefeber et al., 2012; Cevallos-Cevallos et al., 2018; Santos et al., 2020; Chagas-Junior et al., 2021). Yet, thanks to an increased knowledge of the functional roles of strains of individual core species for successful cocoa fermentation courses, a detailed investigation is possible now.

Apart from cocoa curing, subsequent processing of the cured cocoa beans, encompassing roasting of the latter and conching of the chocolate batter, co-defines the organoleptic properties and consumers’ acceptance of the final chocolates, as various physical and chemical transformations during chocolate manufacturing will determine their taste and aroma (Afoakwa et al., 2008; Caligiani et al., 2016; Santander Muñoz et al., 2020; Alvarez-Villagomez et al., 2022). For instance, high temperatures and the consequent dehydration of cocoa as a result of cured cocoa bean roasting reduces the concentrations of volatile organic acids, such as acetic acid, whereas non-volatile organic acids, such as citric acid, lactic acid, succinic acid, and oxalic acid, remain mostly untouched. Consequently, the strong acid taste encountered diminishes (Afoakwa et al., 2008). Furthermore, Maillard reactions are of pivotal importance, as they yield aldehydes (e.g., 2-methyl-1-propanal) and pyrazines (e.g., tetramethylpyrazine) that are associated with typical chocolate notes (Caligiani et al., 2007; Humston et al., 2010; Rodriguez-Campos et al., 2011; Farah et al., 2012; Magi et al., 2012; Kadow et al., 2013). Also, compounds associated with floral and roasted notes arise from Maillard reactions, such as phenethyl acetate and phenethyl alcohol from phenylalanine (Ascrizzi et al., 2017; Moreira et al., 2018; Barišić et al., 2019). During conching of the chocolate batter, for which temperature and time are crucial, remaining moisture and VOCs related to sweet, floral, and fruity notes are removed (Owusu et al., 2012, 2013; Rocha et al., 2017). This could be considered undesirable, but the addition of cocoa butter can compensate for the loss of some of these compounds (Souza and Block, 2018). A decrease of the tetramethylpyrazine and benzaldehyde concentrations and a general increase of the aldehyde concentrations characterizes the conching step (Ziegleder et al., 2005; Bastos et al., 2019). For instance, aldehydes usually increase in relative abundance because of alcohol-to-aldehyde oxidations that are promoted through increased oxygen ingress during conching. Aldehydes can be further oxidized to their corresponding organic acids. Finally, acetate esters decrease in relative abundance, while less volatile esters increase (Clark et al., 2020).

The present study aimed at the investigation of the impact of a strain of each of the yeast species *H. opuntiae* and *P. kudriavzevii*, different from the strains tested before (Díaz-Muñoz et al., 2021, 2023), on Costa Rican Trinitario CFPs and chocolates made from the concomitant cured cocoa beans. It concerned the microbial community and metabolite dynamics of the cocoa pulp-bean mass fermentation, the metabolite dynamics of the drying cocoa beans, and the VOC profiles of the chocolate production steps. To evaluate a possible strain dependency, the data obtained were compared to those from previous SCI CFPs carried out under the same conditions but in different years (Díaz-Muñoz et al., 2021, 2023).

## 2. Materials and methods

### 2.1. Starter culture strains

**Figure 1:**
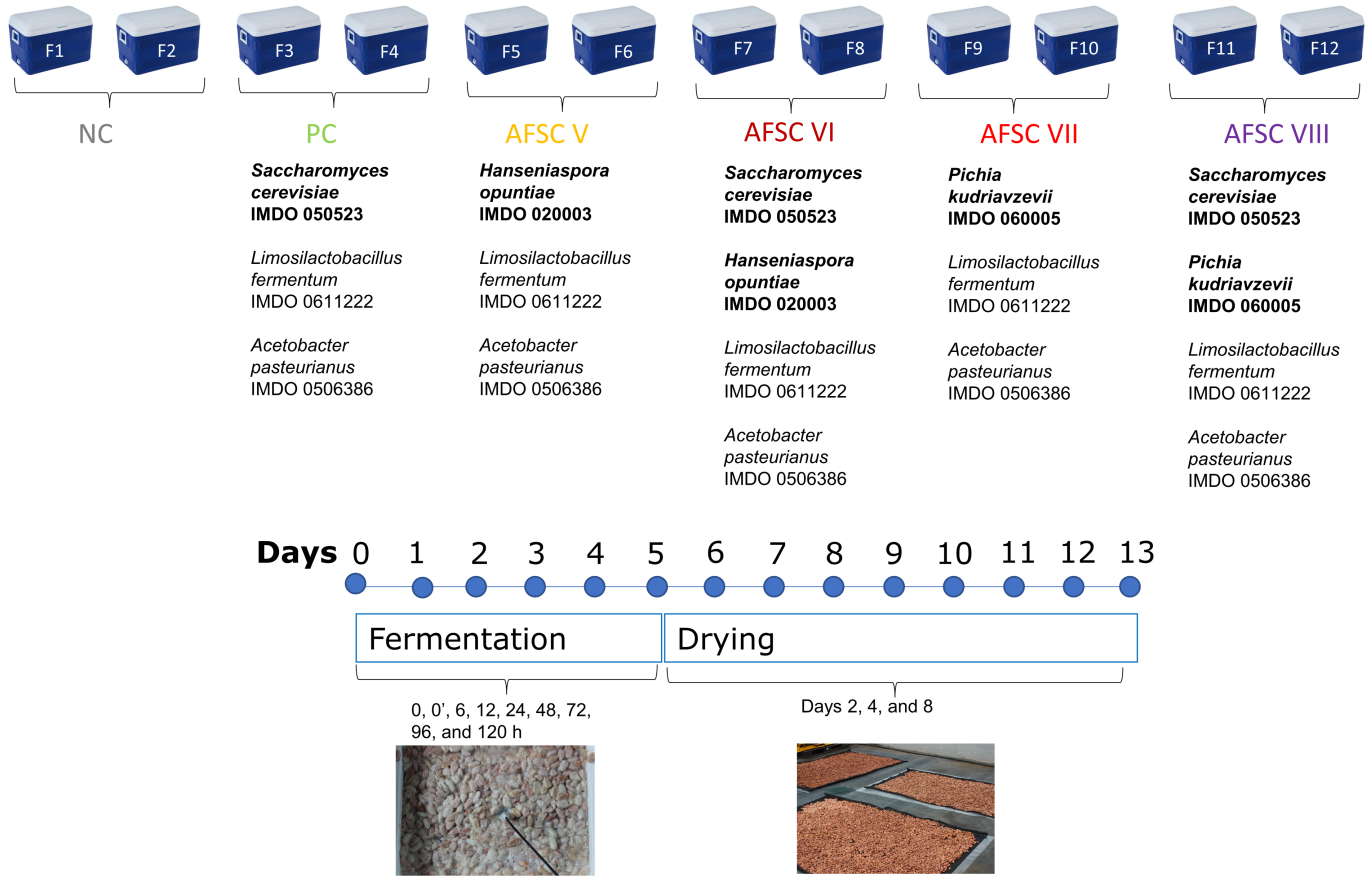
Experimental set-up of two spontaneous cocoa fermentation processes (negative control NC, F1 and F2) and 10 starter culture-initiated cocoa fermentation processes [positive control, F3 and F4; adapted functional starter cultures (AFSCs), AFSC V (F5 and F6), AFSC VI (F7 and F8), AFSC VII (F9 and F10), and AFSC VIII (F11 and F12)], performed in vessels with Trinitario cocoa in Costa Rica.

The microbial strains used throughout this study were the yeasts *S. cerevisiae* IMDO 050523, *H. opuntiae* IMDO 020003, and *P. kudriavzevii* IMDO 060005, the lactic acid bacterium *Limosilactobacillus fermentum* IMDO 0611222, and the acetic acid bacterium *Acetobacter pasteurianus* IMDO 0506386 (Camu et al., 2007; Daniel et al., 2009; Lefeber et al., 2012). Five functional starter culture mixtures were composed with these strains, namely a basic functional starter culture consisting of *S. cerevisiae* IMDO 050523, *Liml. fermentum* IMDO 0611222 and *A. pasteurianus* IMDO 0506386 and used in the positive control (PC) fermentation processes, and four adapted functional starter cultures (AFSCs), which were used in the AFSC V (*H. opuntiae* IMDO 020003, *Liml. fermentum* IMDO 0611222, and *A. pasteurianus* IMDO 0506386), AFSC VI (*S. cerevisiae* IMDO 050523, *H. opuntiae* IMDO 020003, *Liml. fermentum* IMDO 0611222, and *A. pasteurianus* IMDO 0506386), AFSC VII (*P. kudriavzevii* IMDO 060005, *Liml. fermentum* IMDO 0611222, and *A. pasteurianus* IMDO 0506386), and AFSC VIII fermentation processes (*S. cerevisiae* IMDO 050523, *P. kudriavzevii* IMDO 060005, *Liml. fermentum* IMDO 0611222, and *A. pasteurianus* IMDO 0506386; Figure 1). All strains were stored at-80°C in glucose-yeast extract (GY) medium (*S. cerevisiae* IMDO 050523, *H. opuntiae* IMDO 020003, and *P. kudriavzevii* IMDO 060005), de Man-Rogosa-Sharpe (MRS) medium (*Liml. fermentum* IMDO 0611222), or mannitol-yeast extract-peptone (MYP) medium (*A. pasteurianus* IMDO 0506386; Moens et al., 2014). Biomass production and lyophilization of the starter culture strains used was performed as described previously (Díaz-Muñoz et al., 2021).

### 2.2. Cocoa fermentation processes

Six different CFPs were performed in 49-L plastic vessels (Igloo, Katy, Texas, USA) on the campus of the Universidad Nacional de Costa Rica (Heredia, Costa Rica) in November 2019 (transition between wet and dry season; Figure 1). The vessels possessed multiple openings at the bottom for a proper release of the liquefied pulp or sweatings. Every vessel was filled with 36 kg of cocoa pulp-bean mass (without placenta), which was manually scooped out of freshly harvested mature Trinitario cocoa pods from a plantation located in Upala (northern part of Costa Rica). The cocoa pulp-bean mass of every vessel was covered with plantain leaves. Every vessel also contained a lid that remained closed during the first 24 h of fermentation (anaerobic phase). Every fermentation process was performed in duplicate, further referred to as F1 and F2 for the spontaneously inoculated or negative control (NC) fermentation processes, F3 and F4 (PC fermentation processes), F5 and F6 (AFSC V fermentation processes), F7 and F8 (AFSC VI fermentation processes), F9 and F10 (AFSC VII fermentation processes), and F11 and F12 (AFSC VIII fermentation processes). To inoculate the cocoa pulp-bean mass, the lyophilized biomass of the strains was resuspended in saline (0.85%, m/m, NaCl, Merck, Darmstadt, Germany). Inoculation of the cocoa pulp-bean mass with this cell suspension aimed at initial cell densities of 6.0, 6.0, and 5.0 log of colony-forming units (CFU) per g of cocoa pulp-bean mass for yeasts, LAB, and AAB, respectively. All CFPs were performed for 120 h. Ensuing, the cocoa beans were sun-dried for 8 days on a woven polypropylene shade cloth in a greenhouse at the experimental farm Santa Lucia of the Universidad Nacional de Costa Rica.

Along the post-harvest processing chain, samples were taken at time 0 (before inoculation) and time 0′ (after inoculation), after 6, 12, 24, 48, 72, 96, and 120 h of fermentation, and on day 2, 4, and 8 of the drying step, resulting in a total of 106 samples of the fermentation step and 36 samples of the drying step. After each sampling, the cocoa pulp-bean mass in the vessels and the drying cocoa beans on the shade cloth were mixed. Part of the samples withdrawn from the fermenting cocoa pulp-bean mass was used for immediate selective plating for microbial enumeration. The rest of the samples was immediately stored at −20°C for subsequent shipping on dry ice to Belgium for further culture-independent and metabolite target analyses. Also, on average 12.5 kg of the cured cocoa beans of all CFPs, except for F5, F6, F9, and F10 because of moulding during the drying step, were transported to Belgium to make chocolate as described previously (Díaz-Muñoz et al., 2023). During chocolate-making, samples were taken of the cocoa shells, cocoa liquors, conched cocoa liquors, non-tempered chocolates, and final (tempered) chocolates for metabolite target analysis.

### 2.3. Monitoring of temperature, pH, and moisture content

The temperature and pH of the cocoa pulp-bean mass were monitored online during the fermentation step of the different CFPs performed by means of a WTW pH 3110 data logger (Xylem Analytics, Weilheim, Germany). The measurements were automatically stored every 15 min. The internal bean pH was measured on dedicated time points of the fermentation step (72, 96, and 120 h of fermentation) with an Inolab 720 pH meter (Xylem Analytics). Therefore, 10 g of deshelled cocoa beans were crushed and suspended in distilled water for 15 min. The moisture contents of the cocoa beans sampled during the drying step were measured with a Wile Coffee/Cocoa moisture analyser (Farmcomp Oy, Tuusula, Finland).

### 2.4. Off-line monitoring of the microbial community dynamics and species diversity

#### 2.4.1. Selective plating and incubation for colony enumeration

The microbial cell densities at each sampling time point during the fermentation step (seven samples for CFPs F1 and F2, eight samples for the CFPs F3-F12) were determined through a culture-dependent analysis, as described previously (Díaz-Muñoz et al., 2021). Briefly, presumptive counts of yeasts, LAB, and AAB were enumerated by plating on glucose-peptone-yeast extract (YPG) agar medium supplemented with chloramphenicol (200 mg/L, Merck) and incubation at 30°C for 24 h, MRS agar medium supplemented with amphotericin (5 mg/L, Merck) and cycloheximide (200 mg/L, Merck) and incubation at 30°C for 72 h, and modified deoxycholate-mannitol-sorbitol (mDMS) agar medium supplemented with amphotericin (5 mg/L, Merck) and cycloheximide (200 mg/L, Merck) and incubation at 30°C for 120 h, respectively.

#### 2.4.2. Amplicon-based sequencing for microbial community dynamics, microbial species diversity, and starter culture monitoring

Culture-independent microbiological analysis making use of whole-community DNA and applying an ASV approach was performed to estimate the microbial species diversity and community dynamics, and to monitor the growth of the strains of the starter culture mixtures inoculated.

##### 2.4.2.1. Total DNA extraction

Whole-community DNA of cell pellets from 106 cocoa pulp-bean mass samples covering all sampling time points of the fermentation step of all CFPs performed was subjected to a metagenetic analysis, as described previously, with minor modifications (Díaz-Muñoz et al., 2021). For cell pelleting, six cocoa beans with surrounding pulp were mixed with 20 ml of phosphate-buffered saline (PBS; 137 mM NaCl, 2.7 mM KCl, 10 mM Na_2_HPO_4_, 2 mM KH_2_PO_4_, pH 7.0; all chemicals from Merck) in a 50-ml tube by manually shaking for 1 min, followed by shaking on a rotator mixer at 40 rpm for 10 min. Subsequently, the cocoa beans were discarded and more PBS was added until a final volume of 45 ml was obtained. Then, these mixtures were centrifuged at 200 x *g* for 5 min at 4°C, and the supernatants were collected by decantation to separate the coarse materials. The supernatants obtained were subjected to centrifugation at 4,696 x *g* for 20 min at 4°C to pellet the cells. Subsequently, these supernatants were discarded and the cell pellets were resuspended in 5 ml of sorbitol buffer [1.5 M sorbitol (VWR International, Radnor, Pennsylvania, USA), 50 mM Tris base (Calbiochem, San Diego, California, USA), RNase-free water (VWR), pH 8.5], followed by centrifugation at 4,696 x *g* for 10 min at 4°C. These final cell pellets were stored at −20°C until further use.

For actual DNA extraction, once the cell pellets were thawed, an optimized cell lysis protocol was executed, based on a method described previously (Díaz-Muñoz et al., 2021). Briefly, 1,200 μl of yeast cell lysis buffer, 1,000 μl of bacterial cell lysis buffer, 40 μl of sodium dodecyl sulphate (SDS) solution, and 50 μl of proteinase K solution were added (all from Merck). Then, the DNA was directly extracted after applying mechanical disruption and chemical cell lysis steps, using 1,500 μl of a 49.5:49.5:1.0 chloroform-phenol-isoamyl alcohol solution (Sigma-Aldrich, St. Louis, Missouri, USA). Twenty μl of RNAse (Thermo Fischer Scientific, Waltham, Massachusetts, USA) was added and the incubation was extended to 30 min. The DNA purity was checked by means of a NanoDrop ND-2000 spectrophotometer (Thermo Fischer Scientific), and the concentration was assessed using a Qubit fluorometer (Thermo Fisher Scientific).

##### 2.4.2.2. Amplicon-based high-throughput sequencing analysis

###### 2.4.2.2.1. Amplicon generation

Amplification of the full-length 16S rRNA gene of the bacteria was performed with primer pair 27F-1492R (Callahan et al., 2019). Amplification of the ITS1 region of the fungal ribosomal RNA transcribed unit was performed with primer pair BITS1-B58S3 (Bokulich and Mills, 2013). The amplification, PCR clean-up, size selection, library preparation, and Illumina high-throughput sequencing steps were performed as described previously (De Bruyn et al., 2017). The average number of reads per sample was 20,000; rarefaction was done by means of an in-house method. The data obtained have been deposited in the European Nucleotide Archive (ENA/EBI, Hinxton, Cambridgeshire, UK) under accession number PRJEB57747.^1^

###### 2.4.2.2.2. Microbial community and species diversity dynamics

The amplicon-based sequences obtained were processed with the DADA2 package (version dada2_1.20.0; Callahan et al., 2016), and ASVs were inferred as described previously, with minor modifications, encompassing a thorough optimization of the parameters applied (Díaz-Muñoz et al., 2021).

For the 16S rRNA gene forward and reverse amplicons, the following filtering parameters were applied: maxN = 0, maxEE = 2, minLen = 1,000, and maxLen = 1,600. For the ITS1 region forward and reverse amplicons, the parameters maxN = 0, truncQ = 20, maxEE = (1, 1), trimRight = 10, and minLen = 50 were applied. Taxonomy was assigned with the SILVA database (version 138; Quast et al., 2013) for the bacterial ASVs and the UNITE database (version 04.02.2020; Kõljalg et al., 2013) for the fungal ASVs. The fasta headers of the SILVA database were manually reformatted to accommodate the current taxonomy of the *Lactobacillaceae* (Zheng et al., 2020). The species-level taxonomic identification was assigned with a minimum bootstrap value of 80. Only genera with relative abundances above 0.1% in at least one from the 99 samples containing the bacterial amplicons obtained successfully and above 0.5% from the 102 samples containing the fungal amplicons obtained successfully are reported.

###### 2.4.2.2.3. Monitoring of the starter cultures applied

For a more accurate monitoring of the starter culture strains applied, the basic local alignment search tool (BLAST, version 2.2.30; Altschul et al., 1997) was used to align the ASVs obtained and belonging to the species corresponding with the strains used (except for *Hanseniaspora* for which the genus was used), to the known genome sequences of the inoculated strains [*Liml. fermentum* IMDO 0611222 (Verce et al., 2020), *A. pasteurianus* IMDO 0506386 (Illeghems et al., 2013), *S. cerevisiae* IMDO 050523 (Díaz-Muñoz et al., 2022), *H. opuntiae* IMDO 020003 (unpublished), and *P. kudriavzevii* IMDO 060005 (unpublished)] that contained the 16S rRNA gene(s) (bacteria) and ITS1 region (yeasts).

### 2.5. Off-line monitoring of the substrate consumption and metabolite production dynamics

A metabolomic analysis was performed on extracts of the cocoa pulps, the cocoa beans, and the chocolate-making samples. Aqueous and ethyl acetate extracts were prepared as described previously (Díaz-Muñoz et al., 2021). A metabolite target analysis approach for the determination and quantification of non-volatile substrates and metabolites was performed on the aqueous extracts. Volatile substrates and metabolites were quantified in the ethyl acetate extracts. Pure standards were purchased from either Merck or Thermo Fischer Scientific.

#### 2.5.1. Simple carbohydrates and sugar alcohols

Simple carbohydrates (i.e., fructose, glucose, and sucrose) and sugar alcohols (i.e., glycerol, mannitol, and *myo*-inositol) were quantified in the aqueous extracts of all samples, in duplicate, by means of high-performance anion exchange chromatography with pulsed amperometric detection (HPAEC-PAD), using ICS 3000 chromatographs, equipped with a CarboPac PA-20 (simple carbohydrates) and CarboPac MA-1 column (sugar alcohols; Thermo Fisher Scientific), as described previously (Díaz-Muñoz et al., 2021). Quantification was performed by external calibration, including an internal standard [IS; solution of 0.02 g of rhamnose (Merck) per liter of acetonitrile (Thermo Fischer Scientific)].

#### 2.5.2. Ethanol, acetic acid, and acetoin

Ethanol, acetic acid, and acetoin were quantified in the aqueous extracts of all samples, in duplicate, by means of gas chromatography with flame ionization detection (GC-FID), using a Trace 1310 gas chromatograph equipped with an AI 1310 autosampler (Interscience, Breda, The Netherlands), an instant connect flame ionization detector (Interscience), and a Stabilwax-DA column (Restek, Bellefonte, Pennsylvania, USA), as described previously (Díaz-Muñoz et al., 2021), with minor modifications. Samples were injected (0.8 μl) into the column in split mode, applying a split of 40. The injector temperature was 250°C. The oven temperature was programmed as follows: first 60°C for 1.5 min, followed by a temperature increase to 240°C at a rate of 10°C/min, and then held at 240°C for 10 min. The detector temperature was 250°C. Helium (Air Liquide, Louvain-la-Neuve, Belgium) was used as carrier gas at a constant flow rate of 1.0 ml/min, and nitrogen gas (Air Liquide) was used as make-up gas. Quantification was performed by external calibration, including 1-butanol as IS [solution of 750 ml of acetonitrile (Thermo Fischer Scientific), 12 ml of formic acid (Merck), 250 ml of ultrapure water (MilliQ, Merck), and 250 μl of 1-butanol (Merck)].

#### 2.5.3. Organic acids

The concentrations of organic acids (i.e., citric acid, gluconic acid, glucuronic acid, D-lactic acid, L-lactic acid, malic acid, oxalic acid, and succinic acid) were quantified in the aqueous extracts of all samples, in duplicate, by ultra-performance liquid chromatography coupled to triple-quadrupole tandem mass spectrometry (UPLC-MS/MS). An Acquity UPLC system equipped with a HSS T3 column (all organic acids, except for lactic acid; Waters, Milford, Massachusetts, USA) or an Astec Chirobiotic column (D-lactic acid and L-lactic acid; Sigma-Aldrich), coupled to a Quattro Micro tandem mass spectrometer with a ZSpray electrospray ionization source in the negative ionization mode (Waters) was used for all quantifications, as described previously, with minor modifications (Díaz-Muñoz et al., 2021; Decadt et al., 2023). In the case of the lactic acid enantiomers, the flow rate of the mobile phase, encompassing eluent A composed of 33.3 mM ammonium acetate (Merck) in ultrapure water (MilliQ) and eluent B composed of acetonitrile (Thermo Fischer Scientific), was 0.6 ml/min. For elution, the following gradient was applied: 0 to 15 min, isocratic 15% eluent A and 85% eluent B. Quantification was performed by external calibration, including an IS solution [4 mg of salicylic acid (IS; Honeywell Fluka, Morristown, New Jersey, USA) dissolved in 850 ml of acetonitrile (Thermo Fischer Scientific) and 150 ml of ultrapure water (MilliQ) with 0.386 g of ammonium acetate (Merck)]. All samples were microcentrifuged (19,400 × *g* for 15 min at 10°C) and filtered [0.2-μm hydrophylic polytetrafluoroethylene (H-PTFE) Millex filters; Merck] before injection (10 μl) into the column.

#### 2.5.4. Amino acids

Proteinogenic amino acids (i.e., alanine, isoleucine, leucine, phenylalanine, tyrosine, and valine) were quantified in the aqueous extracts of the cocoa beans, in duplicate, by UPLC-MS/MS, using an Acquity UPLC system, equipped with a HSS T3 column and coupled to a Quattro Micro tandem mass spectrometer with a ZSpray electrospray ionization source in the positive ionization mode (Waters). The flow rate of the mobile phase, composed of 5% (v/v) acetonitrile (Thermo Fischer Scientific) with 1 mM formic acid (Merck) and 1 mM pentadecafluorooctanoic acid (PDFOA, Sigma-Aldrich; eluent A) and 90% (v/v) acetonitrile with 1 mM formic acid and 0.5 mM PDFOA (eluent B; Sigma-Aldrich), was 0.23 ml/min. For elution, the following gradient was applied: 0 to 1 min, 99% eluent A and 1% eluent B; 1 to 8 min, 30% eluent A and 70% eluent B; 8 to 10 min, 0% eluent A and 100% eluent B; and 10 to 25 min, 99% eluent A and 1% eluent B. Quantification was performed by external calibration, including an IS solution [8 mg of 2-amino butyric acid (IS) dissolved in 900 ml of ultrapure water (MilliQ) and 1 mM formic acid (Sigma-Aldrich)]. All samples were microcentrifuged (19,400 × *g* for 15 min at 10°C) and filtered (0.2-μm H-PTFE Millex filters, Merck) before injection (10 μl) into the column.

#### 2.5.5. Biogenic amines

Biogenic amines were quantified in the aqueous extracts of the cocoa beans, in duplicate, through UPLC-MS/MS. The analysis was performed with an Acquity UPLC system, equipped with an HSS T3 column, and coupled to a triple-quadrupole tandem mass spectrometer with a ZSpray electrospray ionization source used in positive ionization mode (Waters), as described previously (Van der Veken et al., 2020). Quantification was performed by external calibration.

#### 2.5.6. Volatile organic compounds

##### 2.5.6.1. Sample preparation

The cocoa pulps, cocoa beans during fermentation, and drying cocoa beans of each CFP were ground to fine powders with liquid nitrogen (Air Liquide), as described previously (Díaz-Muñoz et al., 2021). Similarly, fine powders of the chocolate-making samples of the CFPs F1-F4, F7-F8, and F11-F12 were made.

##### 2.5.6.2. Screening

A non-targeted screening through headspace/solid-phase microextraction coupled to gas chromatography with time-of-flight mass spectrometry (HS/SPME-GC-TOF-MS) of the ground cocoa pulp, cocoa beans, and chocolate-making samples was conducted, in triplicate, using a Trace 1300 gas chromatograph (Thermo Fisher Scientific) equipped with a Stabilwax-MS column (Restek), as described previously (Díaz-Muñoz et al., 2021). This metabolite fingerprinting technique served as a selection of VOCs to be quantified.

##### 2.5.6.3. Quantification

Seventy-two VOCs were quantified in the ethyl acetate extracts of the cocoa pulp and cocoa beans and 102 VOCs in those of the chocolate-making samples, in duplicate, with external calibration, by liquid injection gas chromatography coupled to triple-quadrupole tandem mass spectrometry (LI-GC–MS/MS), using a Trace 1300 gas chromatograph equipped with a TriPlus RSH autosampler and a DBwax-etr column (Thermo Fisher Scientific) connected to a TSQ 8000 EVO triple-quadrupole mass spectrometer (Interscience), as described previously (Díaz-Muñoz et al., 2021).

### 2.6. Statistical analysis

Statistical processing of all data was performed with the software RStudio (version 4.0.5; R Studio Team, 2021). Prior to (multiple) pairwise data comparisons, the normality of the data was assessed with a Shapiro–Wilk test. Additionally, the variances were evaluated for equality by means of a *F*-test. The outcome of the aforementioned tests determined the subsequent tests for possible significances, either a Student *t*-test, Wilcoxon rank-sum test, or analysis of variance (ANOVA) test (Stats package, version 4.0.5; R Core Team, 2021). Noteworthy, two approaches to evaluate statistical significances on time-dependent data were performed with the former tests. One approach conducted statistical tests on the absolute values of the data to assess the quantitative nature of the data as such. The other approach focused on the data series obtained by taking the difference between time point x + 1 and x. As such, trends in time courses were evaluated for statistical significance.

Three principal component analyses (PCAs) were performed and plotted using ggplot2 (Coultate, 2009; Wickham, 2016) to visualize the comparison of the VOC compositions quantified according to the source [cocoa pulps, cocoa beans during fermentation, drying cocoa beans, cocoa shells, cocoa liquors, conched cocoa liquors, non-tempered chocolates, and final chocolates], functional starter culture applied, and factor loadings on both PCs. Each PCA was based on a correlation matrix of the concentrations of the VOCs quantified in the cocoa pulps, cocoa beans during fermentation, drying cocoa beans, cocoa shells, cocoa liquors, conched cocoa liquors, non-tempered chocolates, and final chocolates. Prior to performing two-dimensional PCAs, a Scree plot served as the decision criterium to include two PCs solely. Furthermore, heatmaps were generated based on Z-score transformation normalized quantitative data by means of the ComplexHeatmap package (version 2.6.2; Gu et al., 2016). Hierarchical clustering analysis was based on the Ward’s method (clustering method set to “Ward.D2”). The Euclidean distance calculation method was applied to assess the similarity between samples.

Finally, correlations between the microorganisms and metabolites identified throughout the CFPs performed were assessed by constructing correlation matrices with the mixOmics package (version 6.14.1; Rohart et al., 2017).

## 3. Results

### 3.1. pH and temperature patterns

**Figure 2:**
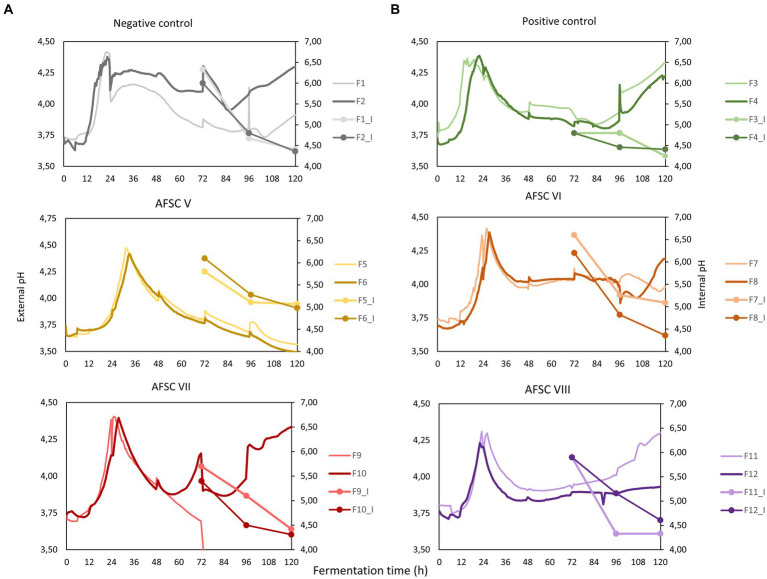
**(A)** Course of external pH (cocoa pulp-bean mass, full lines), internal pH (cocoa beans, connected lines), and **(B)** temperature (cocoa pulp-bean mass) during 120-h cocoa fermentation processes, performed in vessels with Trinitario cocoa in Costa Rica. The type of fermentation process (F1-F12) is as explained in the legend of Figure 1.

The initial pH and temperature of the cocoa pulp-bean mass of the six Costa Rican Trinitario CFPs performed was in the range from 3.7 to 3.8 and from 22.6 to 24.4°C, respectively (Figure 2). The pH and temperature course of these CFPs followed the common pattern of cocoa pulp-bean mass fermentation processes. However, the AFSC V fermentation processes displayed a continuous pH decrease after a pH increase during the anaerobic phase (0–24 h). The final pH and temperature of the fermenting cocoa pulp-bean mass of all CFPs was in the range from 3.5 to 4.3 and from 32.5 to 45.2°C, respectively. Whereas the pH profiles of the AFSC VI fermentation processes were not significantly different compared to the NC (*p* = 0.06), PC (*p* = 0.20), and AFSC VIII (*p* = 0.84) ones, the pH profiles of the other CFPs were significantly different among each other (*p* < 0.05), except for the AFSC VII fermentation processes compared to the NC (*p* = 0.60) and AFSC VI (*p* = 0.44) ones. The temperature profiles of all CFPs were significantly different among each other (*p* < 0.05). None of the biological duplicates of all CFPs performed were significantly different (*p* < 0.05) among each other.

The initial internal bean pH, first measured after 72 h of fermentation, was 6.2, 4.7, 6.0, 6.4, 5.6, and 5.6 for the NC, PC, AFSC V, AFSC VI, AFSC VII, and AFSC VIII fermentation processes, respectively (Figure 2A). This internal bean pH decreased with the fermentation time to final values of 4.3, 4.3, 5.0, 4.7, 4.4, and 4.5, respectively. None of the internal pH courses were significantly different (*p* < 0.05) among each other. However, applying the basic functional starter culture (PC fermentation processes) significantly affected (*p* = 0.03) the internal bean pH compared to the spontaneous fermentation processes (NC). No significant differences (*p* < 0.05) were found among the biological duplicates of the different CFPs performed.

### 3.2. Culture-dependent microbial community dynamics

The initial counts (before inoculation, T0) as well as the dynamics of the counts of the presumptive yeasts, LAB, and AAB were similar to those of CFPs performed previously (Supplementary Figure S1). Concerning the yeast community dynamics, significant differences occurred only in the case of the NC fermentation processes compared to the AFSC VI ones (*p* = 0.0017) and in the case of the PC fermentation processes compared to the AFSC VI (*p* = 0.038) and AFSC VII (*p* = 0.044) ones. Furthermore, the NC fermentation processes were significantly different compared to the AFSC VI ones for both the presumptive LAB (*p* = 0.000031) and AAB (*p* = 0.0011) community dynamics. The biological duplicates of all CFPs performed were not significantly different among each other concerning their microbial community dynamics. Yet, the presumptive yeasts and AAB tended to decline faster toward the end of the F3 fermentation process compared to F4 (PC) and the F11 fermentation process compared to F12 (AFSC VIII; *p* = 0.102).

### 3.3. Amplicon sequence variant approach for culture-independent microbial community dynamics, microbial species diversity, and starter culture monitoring

#### 3.3.1. Microbial community and species diversity dynamics

The full-length 16S rRNA gene and the ITS1 region of the fungal rRNA transcribed unit were used to infer ASVs that allowed a high-resolution taxonomic classification of the bacterial and fungal communities of all CFPs carried out, respectively.

##### 3.3.1.1. Bacteria

**Figure 3:**
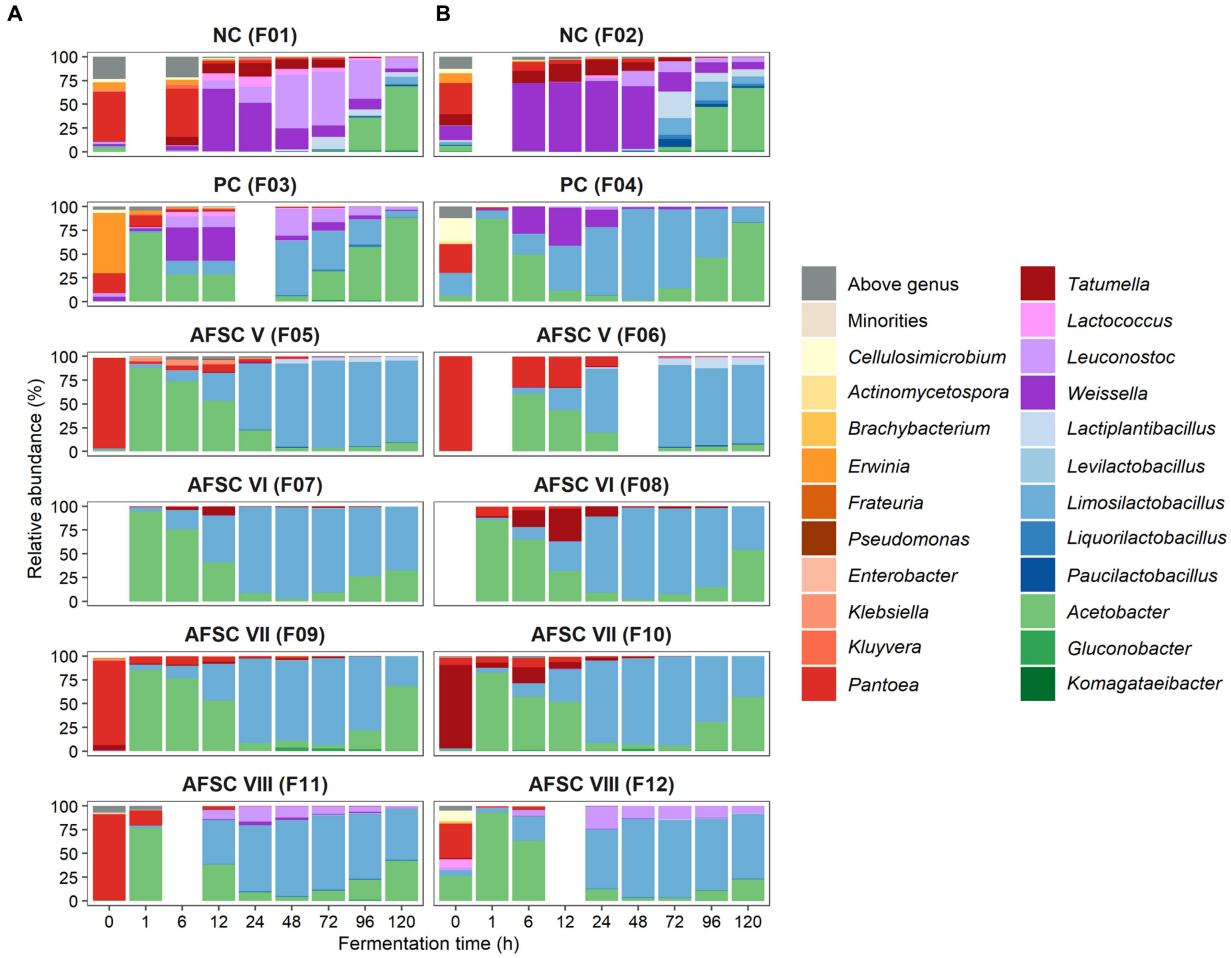
Dynamics of the bacterial **(A)** and fungal diversity **(B)** of 120-h cocoa fermentation processes, performed in vessels with Trinitario cocoa in Costa Rica, expressed as relative abundances based on amplicon sequence variants (ASVs) of the full-length 16S rRNA gene and internal transcribed spacer (ITS1) region, respectively. ASVs that could not be allocated to genus level are represented as ‘Above genus’, whereas communities with a relative abundance below 0.5% are referred to as ‘Others’. The type of fermentation process (F1-F12) is as explained in the legend of Figure 1.

The bacterial communities were predominated by the genera *Acetobacter* and *Limosilactobacillus* for all SCI CFPs performed (Figure 3A). Indeed, the relative abundances of these genera were lower in the NC fermentation processes (0.1–67.7% and 0.0–19.9%, respectively) compared to the SCI CFPs (0.2–94.7% and 0.0–97.1%, respectively). In contrast to the spontaneous fermentation processes, the PC, AFSC VI, AFSC VII, and AFSC VIII fermentation processes showed high relative abundances of the *Acetobacter* genus (F7-F12; 94.7, 85.3, 85.7, 82.0, 76.5, and 92.9%, respectively). Withal, the NC and PC fermentation processes revealed a peak in relative abundance for the *Acetobacter* genus after 120 h of fermentation (F1-F4; 67.7, 65.3, 87.8, and 83.2%, respectively). In contrast, the relative abundance of *Acetobacter* was suppressed at the end of the AFSC V fermentation processes (F5 and F6; 8.7 and 7.0%, respectively) and to a lesser extent for the AFSC VI ones (F7 and F8; 31.3 and 53.4%, respectively). Indeed, in those particular CFPs, *Limosilactobacillus* overruled *Acetobacter* with respect to relative abundance. In the SCI CFPs, *Limosilactobacillus* prevailed from 24 to 72 h of fermentation (relative abundance of 58.1–97.1%). The NC fermentation processes (F1 and F2) only reached moderate relative abundances of *Limosilactobacillus* toward the late fermentation phase (96–120 h) of 7.5 and 19.9%, respectively. Finally, apart from the genera *Acetobacter* and *Limosilactobacillus*, other genera were found among the bacterial communities. In the NC fermentation processes (F1 and F2), also *Weissella*, *Leuconostoc*, *Lactiplantibacillus*, *Pantoea,* and *Tatumella* were represented by noteworthy maximal relative abundances (68.1–74.2%, 56.5–15.9%, 13.0–28.0%, 46.4–37.0%, and 13.9–18.4%, respectively) and to a lesser extent *Liquorilactobacillus* and *Paucilactobacillus*. Moreover, *Weissella* and *Leuconostoc* were represented by moderate relative abundances in the PC fermentation processes (35.6–39.2% and 27.4–5.2%, respectively). Ultimately, the AFSC V fermentation processes contained high maximal relative abundances of the genus *Pantoea* (F5 and F6; 95.2 and 99.3%, respectively). *Tatumella* was present at moderate maximal relative abundances in the AFSC VI (F8) and AFSC VII (F10) fermentation processes (33.7 and 17.0%, respectively). These enterobacterial genera were mostly present at the initial stages of the CFPs and decreased in relative abundance throughout the fermentation courses, especially in the SCI ones. Albeit that the classification at species level for *Enterobacterales* is not always possible due to the high degree of conservation of 16S rRNA genes, *Pantoea rwandensis* (NC and PC), *Pantoea dispersa* (AFSC V, VII, and VIII), *Pantoea ananatis* (AFSC V), *Pantoea stewartii* (AFSC VI), *Tatumella ptyseos* (NC and AFSC V), *Kluyvera ascorbata* (PC), and *Klebsiella michiganensis* (AFSC V) were the main species recovered.

**Figure 4:**
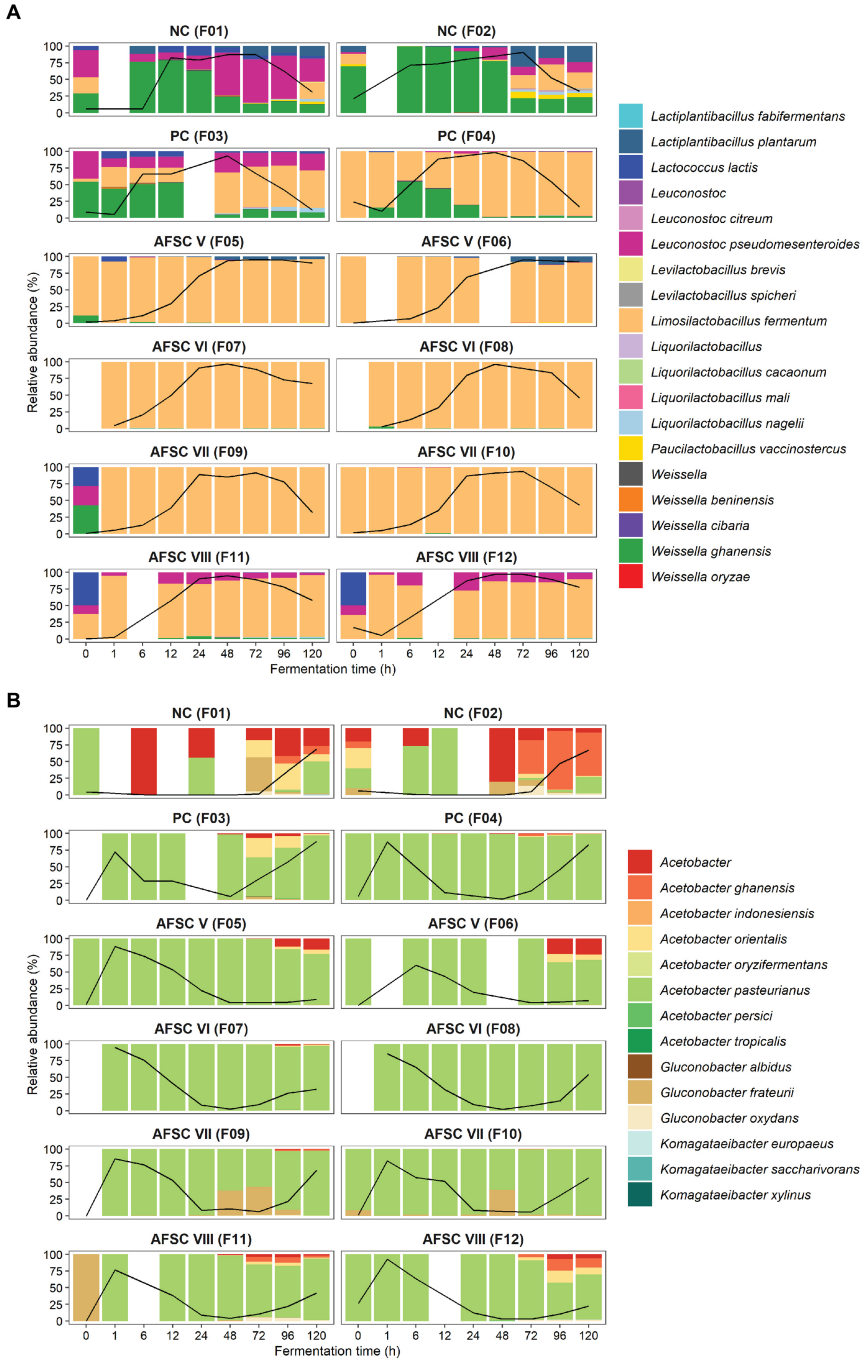
Dynamics of the lactic acid bacterial **(A)** and acetic acid bacterial **(B)** species diversity of 120-h cocoa fermentation processes, performed in vessels with Trinitario cocoa in Costa Rica, expressed as relative abundances based on amplicon sequence variants (ASVs) of the full-length 16S rRNA gene. ASVs that could not be allocated to genus level are represented as ‘Above genus’, whereas communities with a relative abundance below 0.5% are referred to as ‘Others’. The black lines represent the relative abundances of the lactic acid bacterial and acetic acid bacterial communities over the total bacterial communities. The type of fermentation process (F1-F12) is as explained in the legend of Figure 1.

To examine the main bacterial communities in depth, the LAB and AAB species diversities were further investigated (Figures 4A,B). The LAB species diversity of the NC fermentation processes was rather diverse, since no starter culture mixture was added to the initial cocoa pulp-bean mass, and encompassed mainly *Weissella ghanensis*, *Leuconostoc pseudomesenteroides*, and *Lactiplantibacillus plantarum* (F1), and *Lactococcus lactis, Liml. fermentum,* and *Paucilactobacillus vaccinostercus* (F2) as minor species. In contrast, *Liml. fermentum* prevailed during all SCI CFPs, reflecting a successful inoculation of the starter culture mixture. Some of these LAB species were also found in the PC fermentation processes, namely *Leuc. pseudomesenteroides*, *W. ghanensis*, and *Lc. lactis* in the F3 fermentation process and to a lesser extent in the F4 fermentation process. The AFSC VIII fermentation processes contained noteworthy relative abundances of *Leuc. pseudomesenteroides* apart from the inoculated *Liml. fermentum* strain. Concerning the AAB communities, mainly *A. pasteurianus* prevailed over the fermentation course (Figure 4B). Though, *Acetobacter ghanensis*, *Acetobacter orientalis*, and *Acetobacter* sp. were also found at moderate relative abundances in the NC, and to a lesser extent in the PC, AFSC V, and AFSC VIII fermentation processes. *Gluconobacter frateurii* was found at high relative abundances between 48 and 96 h of fermentation in the F9 fermentation process and after 48 h of fermentation in the F10 fermentation process.

##### 3.3.1.2. Yeasts

The most prevalent genera reported for the yeast communities were *Saccharomyces* for the PC fermentation processes (relative abundance of 40.9 to 96.1%)*, Hanseniaspora* for the AFSC V fermentation processes (relative abundance of 41.5 to 99.1%), and *Pichia* for the AFSC VII fermentation processes (relative abundance of 79.8 to 99.8%), whereas the most abundant genera in the AFSC VI and AFSC VIII fermentation processes were the combination of *Saccharomyces* and *Hanseniaspora* and *Saccharomyces* and *Pichia*, respectively (Figure 3B). These findings were in line with the yeast strains inoculated in the respective initial cocoa pulp-bean mass, suggesting a successful inoculation of the starter culture mixture and the ability of the inoculated strains to prevail over the background microbiota. Indeed, before the inoculation with a starter culture mixture, the yeast genera present were more diverse. The lack of a starter culture in the NC fermentation processes resulted in a higher yeast diversity. *Saccharomycotina* (in particular *Dekkera* and *Hyphopichia*) prevailed at high relative abundance during the first 24 h of fermentation. Those yeasts were taken over by *Hanseniaspora, Pichia*, and/or *Saccharomyces*.

Although the ITS1 region spans amplicon fragments ranging from 100 to 400 bp, a species-level identification was performed, albeit with a lower confidence value than for the bacterial species-level identification based on full-length 16S rRNA genes (Figure 3B). Thus, whereas some ASVs could only be assigned at genus level (mainly for *Hanseniaspora*) due to lack of resolution of the ITS1 sequences, others revealed differences between the *Pichia* species present in the NC fermentation processes (*Pichia terricola, P. kudriavzevii,* and *Pichia kluyveri*), the F3 fermentation process (*P. kluyveri*), and the AFSC fermentation processes for which *P. kudriavzevii* IMDO 060005 was inoculated. In the AFSC VI fermentation processes, *Hanseniaspora* and *S. cerevisiae* prevailed at similar relative abundances, given their simultaneous equal inoculation, during the first 24 h of fermentation, followed by an increase of the latter taxon after 48 h of fermentation, indicating its better adaptation to the fermentation conditions. In contrast, *P. kudriavzevii* was found at higher relative abundance (median of 6.87 times higher) than *S. cerevisiae*, from the inoculation until the end of the AFSC VIII fermentation processes, despite their equal inoculation.

#### 3.3.2. Starter culture monitoring

##### 3.3.2.1. Bacteria

**Figure 5:**
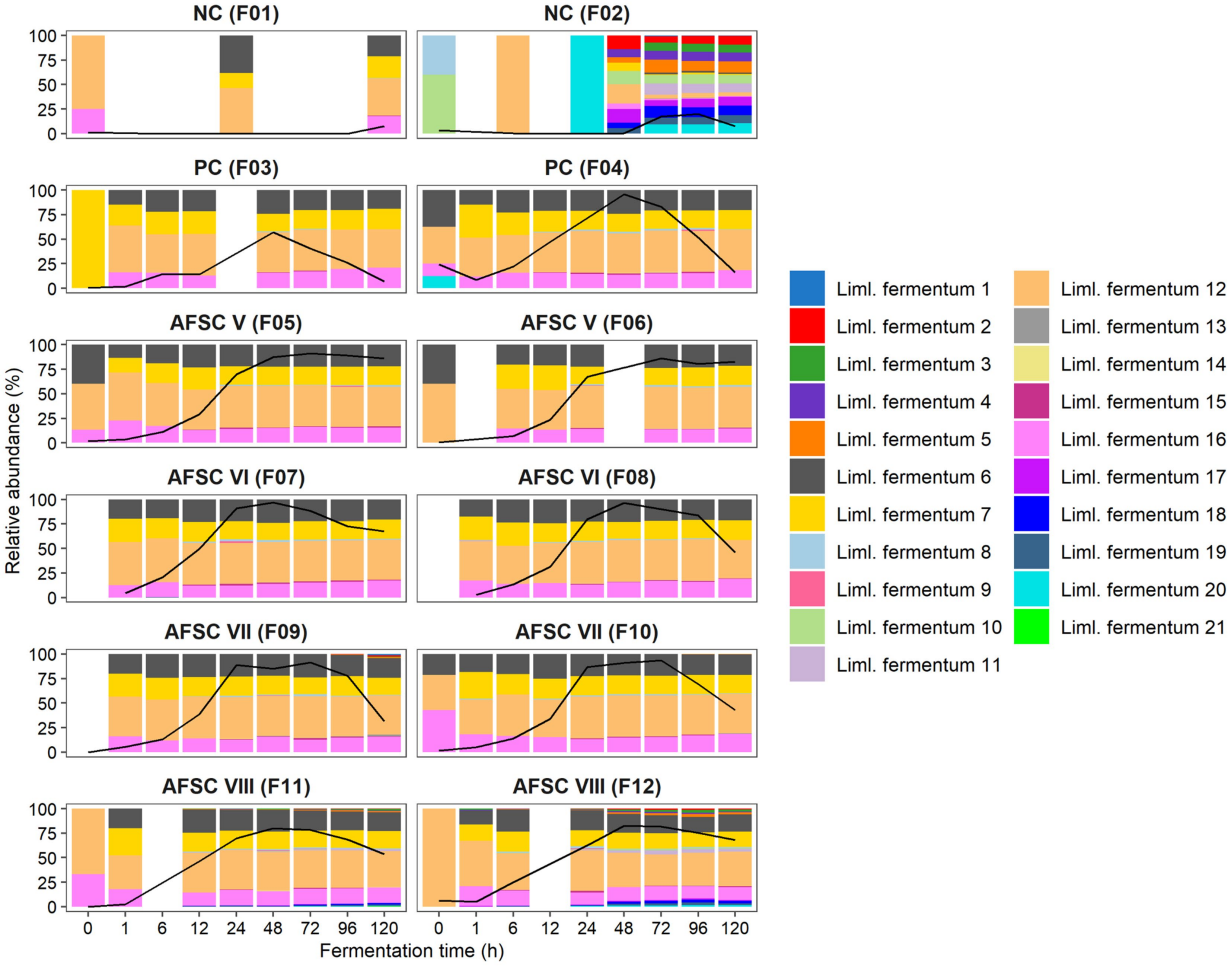
Dynamics of the lactic acid bacterial strain-level diversity of 120-h cocoa fermentation processes, performed in vessels with Trinitario cocoa in Costa Rica, expressed as relative abundances based on amplicon sequence variants (ASVs) of the full-length 16S rRNA gene. The black lines represent the relative abundances of the species *Limosilactobacillus fermentum* in the total bacterial community. The type of fermentation process (F1-F12) is as explained in the legend of Figure 1.

The amplification of the full-length 16S rRNA gene (approximately 1,450 bp) allowed a more accurate identification of the strains present in the fermenting cocoa pulp-bean mass and, hence, a closer monitoring of the starter culture strains inoculated (Figure 5). Therefore, the bacterial ASVs belonging to strains of the inoculated species were examined in detail, namely those of *Liml. fermentum* and *A. pasteurianus*. A total of 21 different *Liml. fermentum* ASVs were inferred for the 12 CFPs performed. Yet, a high reproducibility regarding the ASVs found during all SCI CFPs performed was obtained. It concerned four main ASVs, namely *Liml. fermentum* 6, 7, 12, and 16. Moreover, these ASVs showed 100% identity toward at least one of the contigs of the *Liml. fermentum* IMDO 0611222 genome that covered its 16S rRNA gene. Indeed, the alignment results suggested the presence of five different 16S rRNA gene copies. Thus, the 16S rRNA gene corresponding to the *Liml. fermentum* 12 ASV would be present twice in the genome, since this ASV was present on average 2.26 ± 0.49 times that of the *Liml. fermentum* 6, 7, and 16 ASVs. In contrast, the diversity of ASVs found in one of the spontaneous CFPs (F2) was much higher, consisting of up to 16 different *Liml. fermentum* ASVs after 48 h of fermentation, suggesting that the inoculated strain, *Liml. fermentum* IMDO 0611222, could prevail over the background microbiota in the SCI CFPs. Regarding *A. pasteurianus*, only one ASV was inferred, indicating a low variability in the 16S rRNA genes among *Acetobacter* species, which did not allow to separate putative environmental strains from the inoculated one. However, it was likely that the single *A. pasteurianus* ASV detected in the SCI fermentation processes corresponded with the inoculated strain because of its predominance.

##### 3.3.2.2. Yeasts

**Figure 6:**
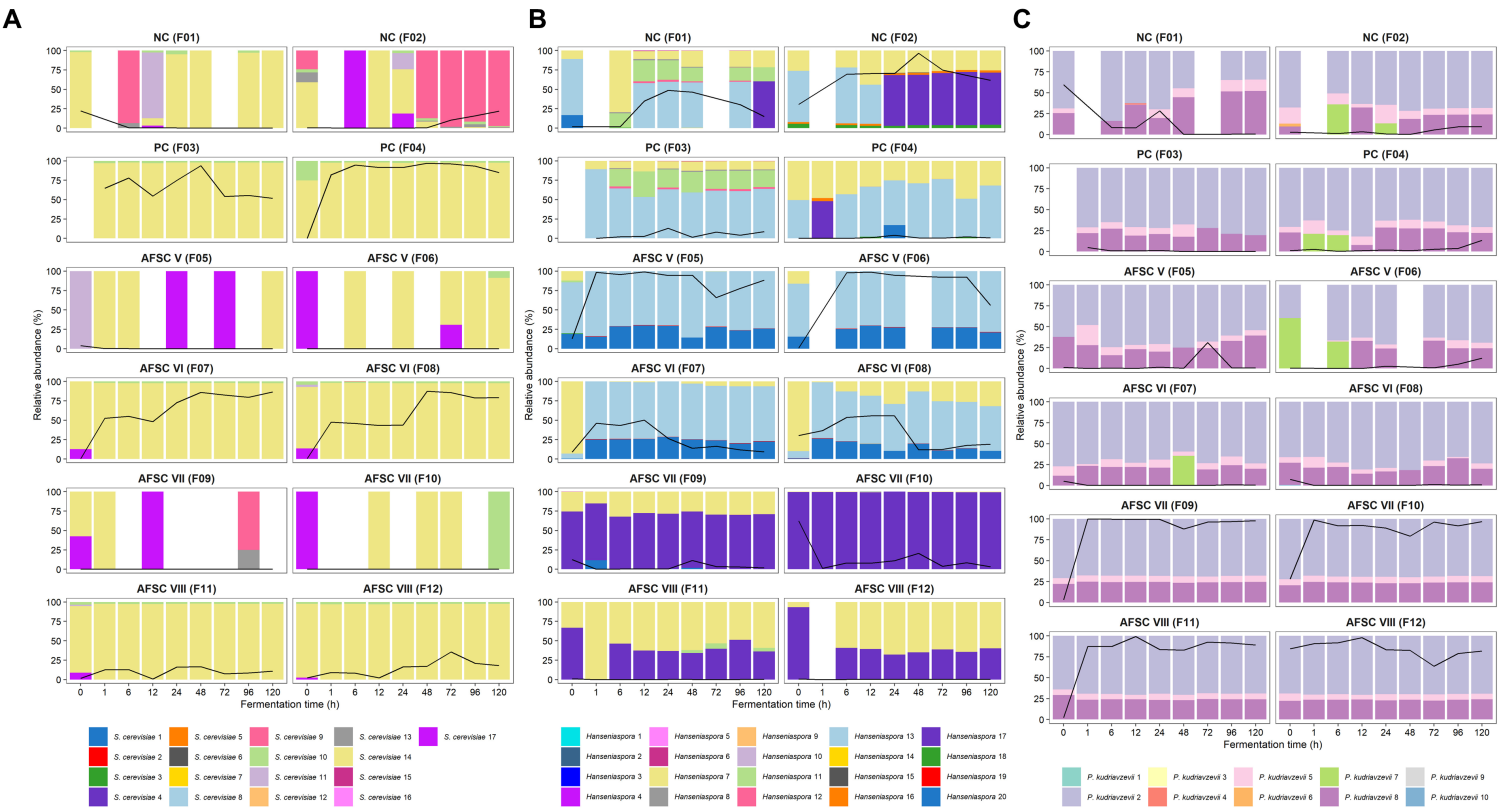
Dynamics of the *Saccharomyces cerevisiae* **(A)**, *Hanseniaspora* **(B)**, and *Pichia kudriavzevii* **(C)** strain-level diversity of 120-h cocoa fermentation processes, performed in vessels with Trinitario cocoa in Costa Rica, expressed as relative abundances based on amplicon sequence variants (ASVs) of the internal transcribed spacer (ITS1) region of the fungal rRNA transcribed unit. The black lines represent the relative abundances of the species *S. cerevisiae* **(A)**, the genus *Hanseniaspora* **(B)**, and the species *P. kudriavzevii* **(C)** in the total fungal communities. The type of fermentation process (F1-F12) is as explained in the legend of Figure 1.

Concerning strain-level identification of the yeasts, 17 different ASVs belonging to *S. cerevisiae* were inferred, with the *S. cerevisiae* 10 and 14 ASVs being present at the highest relative abundances in the CFPs inoculated with *S. cerevisiae* IMDO 050523 (PC, AFSC VI, and AFSC VIII; Figure 6A). Yet, only the *S. cerevisiae* 14 ASV showed 100% identity toward the ITS1 region in the genome of the inoculated *S. cerevisiae* IMDO 050523 strain (Supplementary Table S1). Yet, this ASV was also present in the CFPs that were not inoculated with this starter culture strain. In contrast, the *S. cerevisiae* 9 ASV was the most abundant one in the spontaneous fermentation process F2, indicating that the *S. cerevisiae* population present in the spontaneous CFPs was different from that of the starter culture strain. Regarding *P. kudriavzevii*, 10 different ASVs were found, although mainly three were present at every time point during all CFPs, namely the *P. kudriavzevii* 2, 5, and 8 ASVs (Figure 6B). Among those, only the *P. kudriavzevii* 8 ASV showed 100% identity toward the ITS1 region in the genome of the inoculated *P. kudriavzevii* IMDO 060005 strain (Supplementary Table S2). The presence of a third ASV (*P. kudriavzevii* 5) was not in line with the existence of only two different variants of the ITS1 region in the genome of the starter culture strain. Nevertheless, the appearance of these three ASVs in the same ratios of relative abundance throughout the CFPs suggested that they were part of the genome of the same strain, likely the inoculated one. Since the *P. kudriavzevii* 5 ASV was present in low relative abundance compared to the *P. kudriavzevii* 2 and 8 ASVs, the single nucleotide variant (SNV) that differentiated this ASV from the other two was unnoticed as a real variant by the assembler due to a low coverage (Supplementary Figure S2). The presence of these three ASVs in non-inoculated CFPs suggested that the *P. kudriavzevii* strains that were naturally present contained an identical ITS1 region as the inoculated starter culture strain. Finally, 20 different *Hanseniaspora* ASVs were found, distributed over the 12 CFPs performed (Figure 6C). However, a clear difference in the ASV compositions between the AFSC V and AFSC VI (both inoculated with *H. opuntiae* IMDO 020003) and all other CFPs performed could be shown. Whereas the *Hanseniaspora* 13 and 20 ASVs were the most abundant ones during the AFSC V and AFSC VI fermentation processes, a high diversity was found for the other CFPs performed, with the *Hanseniaspora* 7, 8, 11, 12, 13, and 17 ASVs appearing in different proportions. As was the case for the *P. kudriavzevii* ASVs, the ratio of appearance between the *Hanseniaspora* 13 and 20 ASVs during the SCI CFPs was maintained, from the inoculation with *H. opuntiae* IMDO 020003 until the end of these fermentation processes, suggesting that they were both part of the inoculated strain. Likewise, only one ASV showed 100% homology toward the ITS1 region in the genome of the inoculated *H. opuntiae* IMDO 020003 strain, namely the *Hanseniaspora* 13 ASV (Supplementary Table S3). As the *Hanseniaspora* 13 ASV also appeared in the NC and PC fermentation processes at high relative abundances, the ITS1 fragment was likely shared between different strains of the same species.

### 3.4. Substrate consumption and metabolite production dynamics

#### 3.4.1. Simple carbohydrate consumption

##### 3.4.1.1. Cocoa pulp

A low concentration of sucrose was present in the cocoa pulp in the beginning of the fermentation step, an indication of the use of mature cocoa pods. For both glucose and fructose, high initial concentrations in the range from 92.6 to 127.7 mg/g and from 88.0 to 132.2 mg/g, respectively, were present in the cocoa pulp of all CFPs performed (Supplementary Figure S3A). For all CFPs, both glucose and fructose were consumed by the end of the fermentation step.

No significant differences in the glucose and fructose concentration profiles throughout the entire fermentation courses were found among the CFPs performed. However, the consumption of glucose and fructose was significantly faster (*p* = 0.032) in the SCI CFPs compared to the spontaneous ones. Indeed, in the PC, AFSC VI, AFSC VII, and AFSC VIII fermentation processes, the majority of glucose and fructose was depleted within 48 h of fermentation, whereas the NC fermentation processes needed 96 h of fermentation to reach the same degree of depletion. No other significant differences were found among the different CFPs performed. However, there was a faster glucose consumption tendency, within 48 h of fermentation, in the PC and AFSC VI fermentation processes compared to the NC ones (*p* = 0.12), reflecting the presence of the *S. cerevisiae* starter culture strain in both SCI CFPs. Additionally, when compared to the AFSC V fermentation processes, the PC (*p* = 0.17), AFSC VI (*p* = 0.12), and AFSC VIII (*p* = 0.18) fermentation processes, containing the *S. cerevisiae* starter culture strain, had the tendency to deplete glucose faster. Similarly to the consumption of glucose in the cocoa pulp, fructose showed a faster consumption tendency during the AFSC VI (*p* = 0.19) and AFSC VIII (*p* = 0.16) fermentation processes in comparison with the AFSC V ones.

##### 3.4.1.2. Cocoa beans

Concerning the cocoa beans, the initial glucose and fructose concentrations for all CFPs performed were in the range from 0.8 to 1.4 mg/g and from 1.8 to 2.3 mg/g, respectively (Supplementary Figure S3B). The fructose concentrations in the cocoa beans of all CFPs increased until 48–72 h of fermentation, followed by a decrease toward the end of the fermentation step. The cocoa beans of the AFSC VIII fermentation processes reached significantly lower (*p* = 0.049) fructose concentrations after 48 h of fermentation when compared to those of the AFSC VII ones. Moreover, the cocoa beans of the AFSC VIII fermentation processes showed a tendency toward lower fructose concentrations after 48 h of fermentation in comparison with the AFSC V (*p* = 0.095) and AFSC VI (*p* = 0.097) ones. When the cocoa beans of the NC fermentation processes were compared to those of the AFSC VII ones, the former showed a tendency toward lower fructose concentrations after 48 h of fermentation. Subsequently, at the end of the fermentation step, the concentrations of fructose in the cocoa beans of the AFSC VIII fermentation processes were significantly higher when compared to those of the AFSC V (*p* = 0.013) and AFSC VII (*p* = 0.029) ones. Additionally, the fructose concentrations in the cocoa beans of the PC fermentation processes had a tendency to be lower when compared to those of the AFSC V (*p* = 0.071) and AFSC VIII (*p* = 0.080) ones after 120 h of fermentation. With respect to the glucose concentrations in the cocoa beans, the AFSC VII fermentation processes had significantly higher levels during the first 48 h of fermentation, when compared to the AFSC VIII ones. The cocoa beans of the AFSC V (*p* = 0.095) and AFSC VI (*p* = 0.097) fermentation processes had a tendency toward higher glucose concentrations during the first 48 h of fermentation in comparison with the AFSC VIII ones. Finally, the initial sucrose concentrations of the cocoa beans were in the range from 10.9 to 12.1 mg/g. The sucrose concentrations showed a relatively stable pattern upon fermentation. However, toward the end of the fermentation step, the slight increase of the sucrose concentrations was significantly higher in the NC (*p* = 0.019) and AFSC V (*p* = 0.025) fermentation processes when compared to the AFSC VIII ones and for the AFSC VI fermentation processes in comparison with the AFSC V ones (*p* = 0.048).

After 2 days of drying, the concentrations of glucose, fructose, and sucrose in the cocoa beans were in the range from 1.4 to 3.7 mg/g, from 1.0 to 3.0 mg/g, and from 3.6 to 10.4 mg/g, respectively (Supplementary Figure S3C). The concentrations of glucose and fructose were significantly lower (*p* < 0.05) in the drying beans originating from the AFSC V fermentation processes compared to all the other ones. Concerning the concentrations of sucrose, those of the drying beans from the AFSC V fermentation processes were significantly higher (*p* < 0.05) than the other ones.

##### 3.4.1.3. Chocolate-making samples

The concentrations of glucose, fructose, and sucrose in the cocoa shells, the cocoa liquors, and the conched cocoa liquors were not significantly different (*p* < 0.05) among each other (Supplementary Table S4). Furthermore, the concentrations of these carbohydrates in the non-tempered chocolates and the final chocolates significantly differed from each other as well as from the cocoa shells, cocoa liquors, and conched cocoa liquors (*p* < 0.05). However, the nature of the CFPs had no significant effect on the carbohydrate concentrations in any of the chocolate-making samples.

#### 3.4.2. Sugar alcohol production

##### 3.4.2.1. Cocoa pulp

Initially, glycerol was not present in the cocoa pulp of any of the CFPs examined. However, during fermentation, glycerol was produced, which resulted in final maximal concentrations ranging from 1.8 to 5.5 mg/g of cocoa pulp (Supplementary Figure S4A). No mannitol was found in the beginning of any CFP. Upon fermentation, maximal concentrations of mannitol were quantified at the end of the fermentation step, ranging from 3.3 to 11.2 mg/g. *Myo*-inositol was found in the cocoa pulps of all CFPs. Its concentration showed a constant pattern with regard to the fermentation time, ranging from 0.3 to 0.5 mg/g initially and from 0.4 to 0.8 mg/g at the end of the fermentation step.

The concentration profiles of the entire fermentation courses were not significantly different among each other concerning glycerol. However, the concentrations showed a tendency to be higher in the AFSC VIII fermentation processes (*p* = 0.07) compared to the NC ones. Moreover, significantly more (*p* < 0.05) glycerol was produced within 48 h of fermentation in all SCI CFPs in comparison with the NC ones (*p* < 0.05). Further, the AFSC VII fermentation processes had significantly higher mannitol concentrations during the entire fermentation course (*p* < 0.05). At the end of the fermentation step, more mannitol (*p* < 0.05) was formed in the AFSC V fermentation processes compared to the other SCI CFPs. No significant differences (*p* < 0.05) were found for the *myo*-inositol concentrations.

##### 3.4.2.2. Cocoa beans

The patterns of the concentrations of glycerol, mannitol, and *myo*-inositol in the cocoa beans were comparable with the cocoa pulps (Supplementary Figure S4B). At the start of all CFPs, no glycerol and mannitol were found in the cocoa beans. At the end of the fermentation step, maximal concentrations of both glycerol and mannitol were found, ranging from 0.0 to 3.0 mg/g and from 0.2 to 0.4 mg/g, respectively. The concentration profiles of *myo*-inositol were steadily throughout all CFPs.

Both the glycerol and *myo*-inositol concentrations were not significantly different among any of the CFPs. However, at the end of the fermentation step, significantly more mannitol was formed in the AFSC VII fermentation processes when compared to the NC and AFSC VI ones (*p* < 0.05), and less in the NC ones (*p* < 0.05) in comparison with the PC, AFSC V, and AFSC VIII fermentation processes. Higher concentrations were found in the AFSC VIII fermentation processes in comparison with all other SCI CFPs (*p* < 0.05), with the exception of AFSC VII that produced more mannitol relative to AFSC VIII (*p* < 0.05).

The drying beans showed a decrease in concentration for all sugar alcohols as a function of the drying time (Supplementary Figure S4C). The concentrations of glycerol, mannitol, and *myo*-inositol were in the range from 0.1 to 0.2 mg/g, from 0.0 to 1.0 mg/g, and from 0.0 to 0.2 mg/g, respectively. The drying beans of the AFSC V fermentation processes contained significantly lower glycerol concentrations compared to the other SCI CFPs (*p* < 0.05). The concentrations of mannitol in the drying beans were significantly higher in the AFSC VII fermentation processes compared to all the other ones.

##### 3.4.2.3. Chocolate-making samples

The glycerol, mannitol, and *myo*-inositol concentrations in the cocoa shells were significantly different (*p* < 0.05) compared to the other chocolate-making samples (Supplementary Table S4). Furthermore, solely the concentrations of mannitol and glycerol were significantly higher (*p* < 0.05) in the cocoa shells of the SCI CFPs compared to the NC ones.

#### 3.4.3. Ethanol and acetic acid production

##### 3.4.3.1. Cocoa pulp

At the start of the fermentation step, no ethanol nor acetic acid were found in the cocoa pulps of any of the CFPs performed (Supplementary Figure S5A). Upon the fermentation course, ethanol was produced, reaching maximal concentrations in the range from 19.7 to 41.0 mg/g after 48–72 h of fermentation. Also, acetic acid peaked at the end of the fermentation step, topping from 12.6 to 36.4 mg/g after 96–120 h of fermentation. None of the entire ethanol concentration profiles were significantly different among each other. However, after 48 h of fermentation, the ethanol concentrations were significantly higher (*p* < 0.05) in the PC fermentation processes compared to the NC, AFSC V, and AFSC VII ones. With respect to the AFSC fermentation processes, the AFSC V ones contained significantly less (*p* < 0.05) ethanol after 48 h of fermentation compared to the AFSC VIII ones. The concentration profiles of acetic acid in the cocoa pulps showed a tendency to be higher (*p* = 0.16) in the PC fermentation processes compared to the other ones. Furthermore, at the end of the fermentation step, the concentrations of acetic acid in the PC fermentation processes were significantly higher (*p* < 0.05) when compared to the NC, AFSC V, AFSC VII, and AFSC VIII ones.

##### 3.4.3.2. Cocoa beans

Initially, no ethanol and acetic acid were present in the cocoa beans of any of the CFPs performed (Supplementary Figure S5B). Upon fermentation, the ethanol and acetic acid concentrations increased to maximal values after 72 and 120 h of fermentation, respectively. No significant differences (*p* < 0.05) were found among the CFPs performed. However, at the end of the fermentation step, the acetic acid concentrations tended to be lower (*p* = 0.09) in the AFSC V fermentation processes compared to the AFSC VIII ones. The acetic acid concentrations showed a decreasing trend according to the drying time, whereas those of ethanol showed a steady pattern (Supplementary Figure S5C). The acetic acid concentration profiles of the drying beans of the AFSC V fermentation processes were significantly lower (*p* < 0.05) in comparison to the ones of the other AFSC fermentation processes.

##### 3.4.3.3. Chocolate-making samples

Acetic acid was present in all chocolate-making samples, whereas ethanol and acetoin were absent (Supplementary Table S4). The acetic acid concentrations were significantly higher (*p* < 0.05) in the cocoa shells compared to the other samples. However, no significant differences could be found with respect to the addition of a starter culture.

#### 3.4.4. Organic acid production

##### 3.4.4.1. Cocoa pulp

Only citric acid (ranging from 18.4 to 22.1 mg/g), gluconic acid (ranging from 0.1 to 0.6 mg/g), malic acid (ranging from 2.4 to 3.4 mg/g), glucuronic acid (ranging from 0.2 to 0.5 mg/g), and, to a lesser extent, succinic acid (ranging from 0.0 to 0.1 mg/g) were present in the cocoa pulps at the start of the CFPs performed (Supplementary Figure S6A). Upon fermentation, citric acid and malic acid were depleted within 48 h in all CFPs. However, at the end of all CFPs, except for AFSC V, the citric acid concentrations again increased. The concentrations of D-lactic acid, L-lactic acid, gluconic acid, glucuronic acid, oxalic acid, and succinic acid (slightly) increased during fermentation.

After 48 h of fermentation, the AFSC VIII fermentation processes revealed significantly higher (*p* < 0.05) concentrations of D-lactic acid and L-lactic acid in comparison with the NC and PC ones. Overall, the concentration profiles of D-lactic acid-and L-lactic acid were higher in the AFSC VIII fermentation processes when compared to the NC, PC, and AFSC VI ones (*p* = 0.08, *p* = 0.10, and *p* = 0.13, respectively). After 72 h of fermentation, the concentrations of oxalic acid were significantly higher (*p* < 0.05) in the AFSC V fermentation processes in comparison to the NC, PC, and AFSC VII ones. Moreover, the PC fermentation processes showed lower (*p* < 0.05) oxalic acid concentrations after 72 h of fermentation when compared to the NC, AFSC V, AFSC VI, and AFSC VII ones. Concerning the concentration profiles of gluconic acid, those of the AFSC VIII fermentation processes were significantly lower (*p* < 0.05) than those of the NC ones. Also, the oxalic acid concentrations of the AFSC VII fermentation processes were higher relative to the PC ones (*p* < 0.05). The malic acid concentrations were not exhausted after 24 h of fermentation in the AFSC V fermentation processes and were thus higher (*p* < 0.05) compared to the NC, PC, and the other AFSC ones, except for AFSC VII. Finally, no significant differences among the CFPs were identified for the concentrations of glucuronic acid and succinic acid, albeit that those of glucuronic acid tended to be higher (*p* = 0.06) in the AFSC VII fermentation processes compared to the AFSC VIII ones.

##### 3.4.4.2. Cocoa beans

Concerning the cocoa beans during fermentation and the drying beans, no significant differences were found in any of the CFPs for any of the organic acids targeted (Supplementary Figures S6B,C).

##### 3.4.4.3. Chocolate-making samples

The concentrations of the above-mentioned organic acids significantly differed (*p* < 0.05) in the cocoa shells compared to the other chocolate-making samples (Supplementary Table S4). None of the CFPs showed a significant impact on any of the organic acid concentrations measured in any of the chocolate-making samples examined.

#### 3.4.5. Amino acid and biogenic amine production in the cocoa beans

Only the cocoa bean concentrations of alanine and tyrosine showed increasing trends with fermentation time, ranging from 0.1 to 0.4 mg/g and from 0.1 to 0.3 mg/g, respectively (Supplementary Figure S7A). The concentrations of the other amino acids quantified, namely isoleucine, leucine, phenylalanine and valine, remained constant (ranging from 0.0 to 0.1 mg/g of cocoa beans). No significant differences could be revealed.

No biogenic amines could be quantified in the cocoa beans at the start of the fermentation step in any of the CFPs examined (Supplementary Figure S7B). The concentrations of tryptamine remained constant with fermentation time, whereas those of tyramine increased. The concentrations ranged from 0.0 to 5.2 mg/g and from 0.0 to 27.9 mg/g, respectively. The overall concentration profile of tryptamine was significantly higher (*p* < 0.05) in the NC fermentation processes compared to all AFSC ones. Additionally, the AFSC VII fermentation processes contained less (*p* < 0.05) tryptamine relative to the PC and AFSC V ones. At the end of the fermentation step, more tyramine was found in the AFSC V fermentation processes (*p* < 0.05) compared to the PC and the other AFSC ones. Further, less tyramine was present in the cocoa beans of the AFSC VIII fermentation processes (*p* < 0.05) relative to those of the AFSC VI and AFSC VII ones at the end of the fermentation step.

#### 3.4.6. Volatile organic compound production

##### 3.4.6.1. Screening and quantification

**Figure 7:**
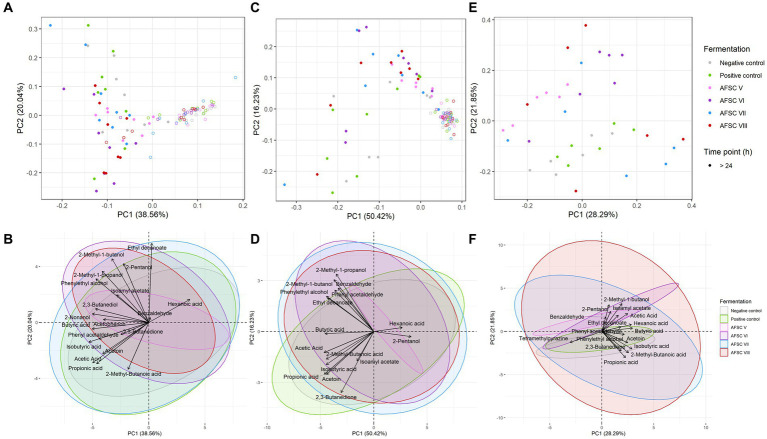
Principal component analysis (PCA) performed on the concentrations of 19 volatile organic compounds (VOCs) in the cocoa pulps **(A)**, 16 VOCs in the cocoa beans during fermentation **(B)**, and 17 VOCs in the drying cocoa beans **(C),** quantified by liquid injection gas chromatography with triple-quadrupole tandem mass spectrometry (LI-GC–MS/MS) during the fermentation phase of twelve 120-h Costa Rican Trinitario cocoa fermentation processes carried out in vessels, followed by eight days of drying. The legend is as explained in Figure 1. Visualization of the influence of dedicated VOCs **(D–F)**.

After HS/SPME-GC-TOF-MS fingerprinting of the cocoa pulps, the cocoa beans during fermentation, the drying cocoa beans, and the chocolate-making samples of all CFPs examined (data not shown), 31 out of 72 VOCs were included for a quantitative LI-GC–MS/MS analysis. Typical metabolites produced by the fermentative microbiota were higher alcohols (e.g., 2-methyl-1-butanol, 2-nonanol, and phenethyl alcohol) and esters (e.g., ethyl decanoate and isoamyl acetate; Figure 7). Noteworthy, tetramethylpyrazine was found and quantified in the drying cocoa beans (Figure 7F).

##### 3.4.6.2. PCAs and heatmaps concerning sample source, fermentation time, and type of fermentation process

**Figure 8:**
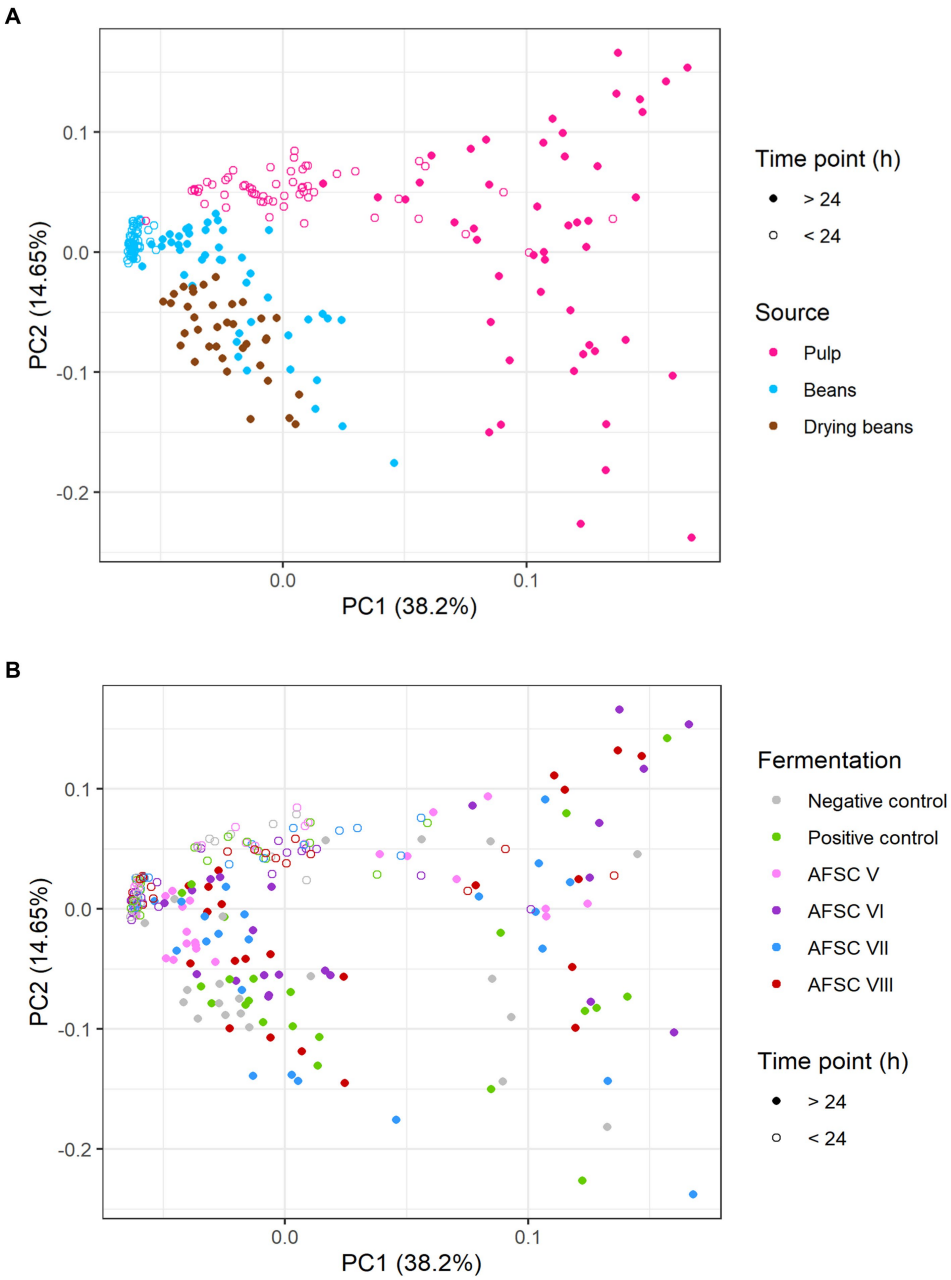
**(A)** Principal component analysis (PCA) performed on the concentrations of 19 volatile organic compounds (VOCs) in the cocoa pulps and 16 VOCs in the cocoa beans, quantified by liquid injection gas chromatography with triple-quadrupole tandem mass spectrometry (LI-GC–MS/MS) during the fermentation and drying phases of twelve 120-h Costa Rican Trinitario cocoa fermentation processes carried out in vessels, followed by eight days of drying. The influence of the sample source (cocoa pulp, cocoa beans during fermentation, and drying cocoa beans) is visualized in pink, blue, and brown, respectively. Samples that were fermented less than 24 h are indicated by open circles, those that were fermented longer are indicated by closed circles. **(B)** PCA on the same data set as (A) to visualize the influence of the type of fermentation process (legend as in Figure 1); the negative control, positive control, adapted functional starter culture (AFSC) V, VI, VII, and VIII fermentation processes are represented in gray, green, pink, purple, blue, and red, respectively.

Based on a minimal presence in two out of three replicates of the samples subjected to the non-targeted screening, a PCA was performed on the quantified VOCs (Figure 8). Overall, 52.85% of the total variance was explained for the cocoa pulps, cocoa beans during fermentation, and drying cocoa beans. Distinctive clusters were obtained according to the source and fermentation time (Figure 8A). Indeed, higher scores on PC1 were found for the samples belonging to the cocoa pulps, especially after 24 h of fermentation, indicating that more differences in VOC composition occurred after the anaerobic fermentation phase. Additionally, the PC1 scores for the cocoa beans during fermentation and the drying cocoa beans were lower compared to the cocoa pulps, whereas the scores on PC2 were more centred. When visualizing the different CFPs with a PCA on the same quantitative data, at first sight no distinctive clusters could be identified according to the starter cultures applied (Figure 8B). However, more diversification in the VOC compositions occurred in the cocoa pulps compared to the cocoa beans during fermentation and the drying cocoa beans. The AFSCs scored more pronounced on both PC1 and PC2 for the cocoa pulps, which was an indication for a higher formation or depletion of certain VOCs. Moreover, the AFSC V fermentation processes (containing the *H. opuntiae* strain as the sole yeast starter culture) scored less pronounced on both PC1 and PC2 according to the fermentation time. Both PC1 and PC2 revealed different scores according to the source and the type of CFP. However, the VOCs that impacted those PCs reflected a desirable flavour potential.

For a more in-depth-analysis, the quantified VOCs of the cocoa pulps, cocoa beans during fermentation, and drying cocoa beans were subjected to different PCAs (Figure 7). A majority of the sample data corresponding to the AFSC-initiated CFPs clustered toward high PC2 scores. The PCA on the quantified cocoa pulp VOCs explained 58.60% of the total variance (Figure 7A). After 24 h of fermentation, the PC scores of all CFPs increased. The NC and PC fermentation processes (indicative for increased PC2 scores) could be distinguished from the AFSC fermentation processes, with the exception of AFSC V. The NC, PC, and AFSC VII fermentation processes covered mainly organic acids. Furthermore, the highest impacts of the VOCs in general were toward the end of the CFPs, as indicated by the time clusters. The PCA dealing with the cocoa beans during fermentation indicated no substantial variance explained by the anaerobic phase (first 24 h of fermentation; Figure 7B). In total, 66.65% of the variance was explained by this PCA. In contrast, the NC and PC fermentation processes had the lowest PC2 scores. As reported above for the cocoa pulps, the cocoa beans linked to the AFSC V fermentation processes were less spread over the PCA compared to the other ones. A PCA on the quantitative data of the drying cocoa beans explained 50.14% of the total variance (Figure 7C). The PC and NC fermentation processes revealed rather negative scores on PC2, whereas the AFSC V and AFSC VI fermentation processes scored rather positive. The clusters of the AFSC VII and AFSC VIII fermentation processes were more randomized, containing both positive and negative scores on PC2.

The same PCA on the sample data of the cocoa pulps (Figure 7A), cocoa beans during fermentation (Figure 7B), and drying cocoa beans (Figure 7C) could be visually represented differently to emphasize the VOC compositions themselves (Figures 7D–F). For the cocoa pulps, the AFSC V fermentation processes contained the least VOCs (Figure 7D). Moreover, cocoa-related compounds, such as higher alcohols and esters (e.g., phenylethyl alcohol and isoamyl acetate, respectively), were mostly found in the AFSC VI and AFSC VIII fermentation processes, which were inoculated with a strain of the yeast species *S. cerevisiae*. Similarly, the different cocoa bean clusters of the CFPs were less different from each other when compared to the cocoa pulps (Figure 7E). Additionally, the NC and PC fermentation processes were linked with organic acids (e.g., isobutyric acid) and the key ester isoamyl acetate. The majority of the higher alcohols (e.g., 2-methyl-1-butanol) and esters (e.g., ethyl decanoate) were associated with the AFSC VI fermentation processes. However, the high overlap with the AFSC VII and AFSC VIII fermentation processes led to the association of AFSC CFPs in general (except for AFSC V), possessing other yeast species than *S. cerevisiae*, to higher alcohols and esters. Generally, no distinctive compound classes could be assigned to the drying cocoa beans of a specific CFP, given the randomized nature of the clusters (Figure 7F).

**Figure 9:**
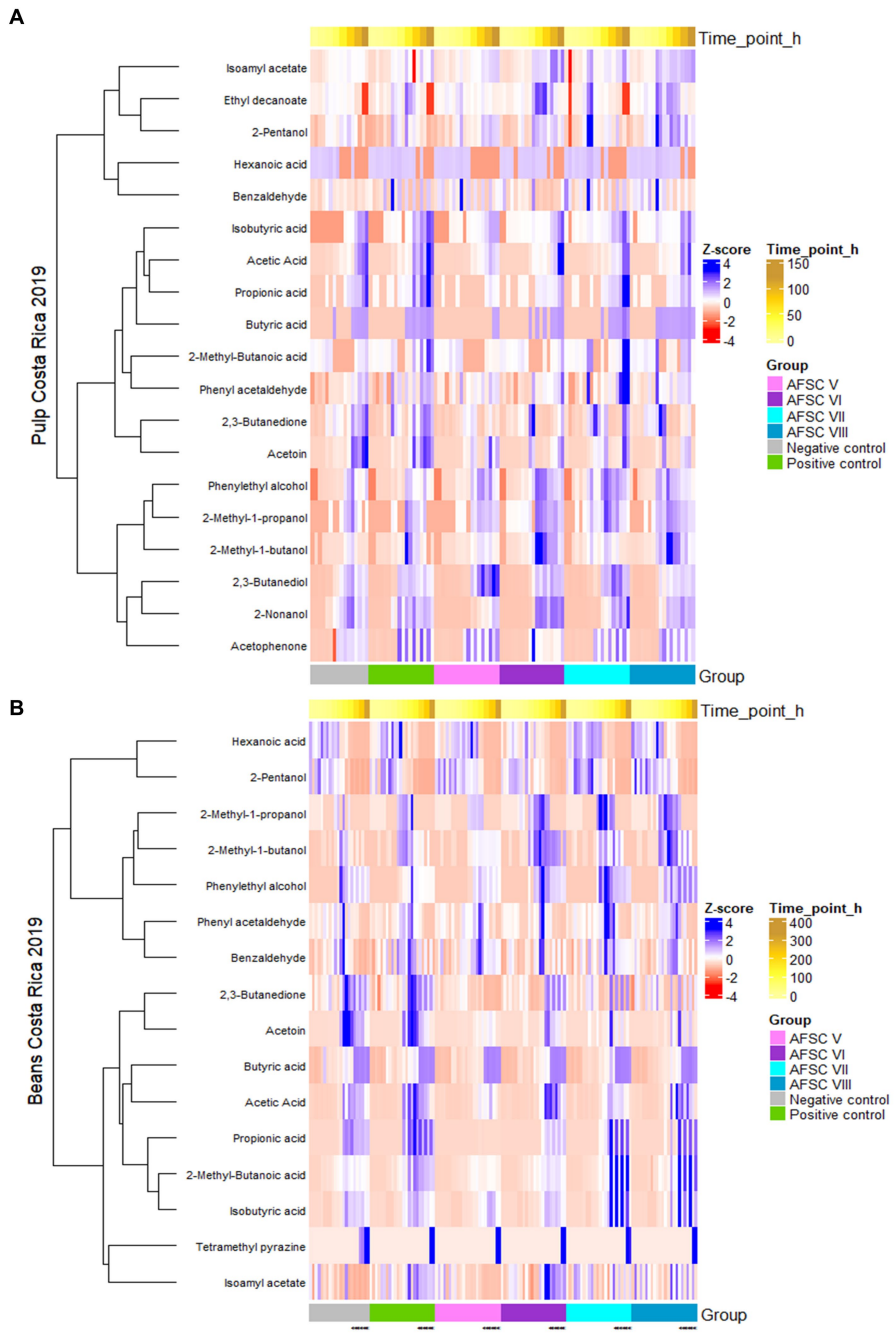
Hierarchical clustering analysis performed on the concentrations of 19 volatile organic compounds (VOCs) in the cocoa pulps **(A)**, 16 VOCs in the cocoa beans during fermentation **(B)**, and 17 VOCs in the drying cocoa beans **(C)**, quantified by liquid injection gas chromatography with triple-quadrupole tandem mass spectrometry (LI-GC–MS/MS) during the fermentation and drying phases of twelve 120-h Costa Rican Trinitario cocoa fermentation processes carried out in vessels, followed by eight days of drying. All VOC concentrations were normalized as *Z*-scores.

All statements mentioned above were also confirmed through a heatmap analysis (Figure 9). For instance, the rather poor dynamics of VOCs in the AFSC V fermentation processes compared to the other AFSC, NC, and PC ones were also visible in the corresponding heatmap. Further, the heatmap analysis of the cocoa bean samples pointed out that also isoamyl acetate seemed to be relevant in the AFSC VI, AFSC VII, and AFSC VIII fermentation processes (Figure 9B).

##### 3.4.6.3. Chocolate-making samples

**Figure 10:**
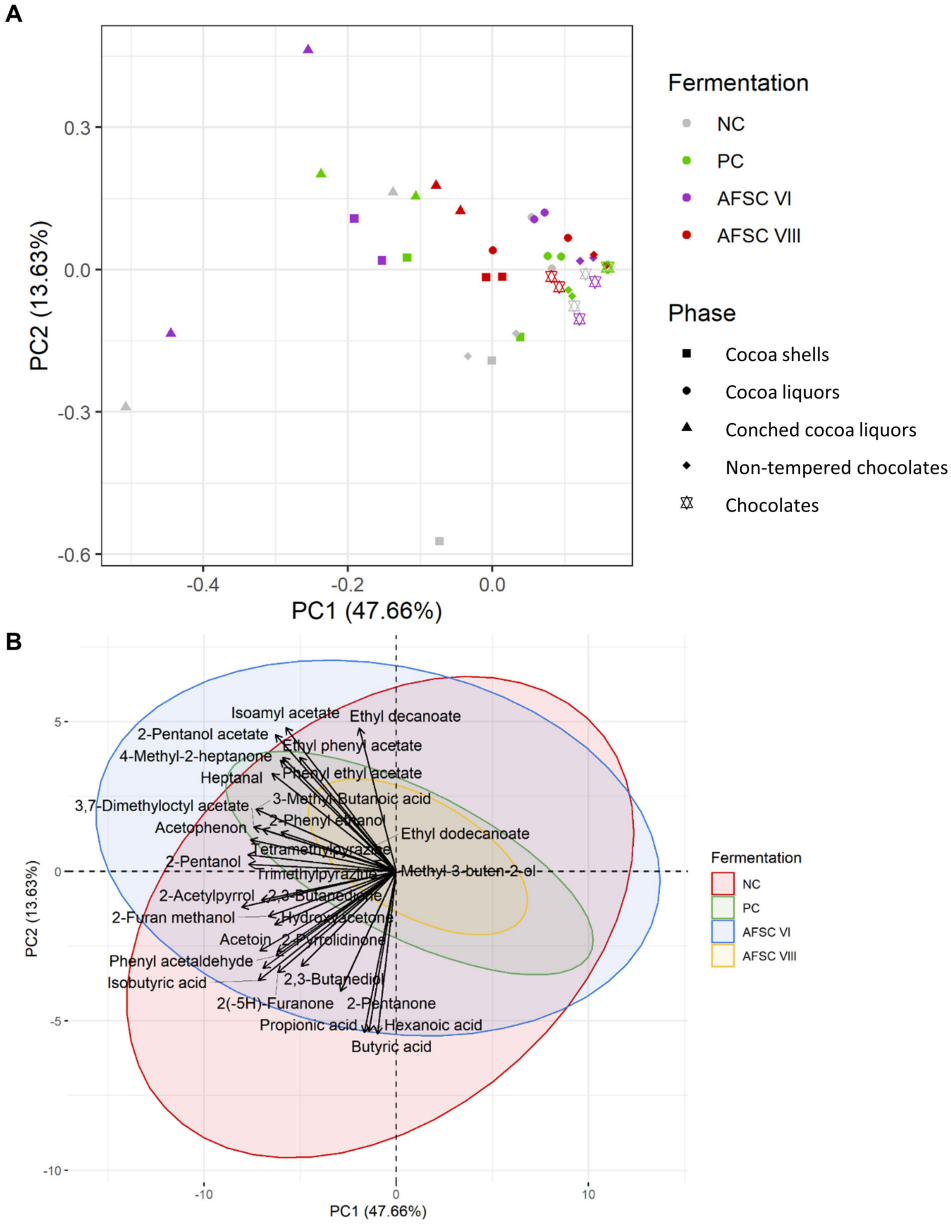
**(A)** Principal component analysis (PCA) performed on the concentrations of 31 volatile organic compounds (VOCs) in the chocolate-making samples, quantified by liquid injection gas chromatography with triple-quadrupole tandem mass spectrometry (LI-GC–MS/MS), and corresponding with twelve 120-h Costa Rican Trinitario cocoa fermentation processes carried out in vessels, followed by eight days of drying and common cocoa processing for chocolate production. The legend is as explained in Figure 1. **(B)** Visualization of the influence of dedicated VOCs. The influence of the type of fermentation process; the negative control, positive control, and adapted functional starter culture (AFSC) VI and VIII fermentation processes are represented in red, green, blue, and yellow, respectively.

The fingerprinting of the chocolate-making samples revealed 102 VOCs, of which 31 VOCs could be quantified. In addition to VOCs characteristic for fermentation and drying, Maillard-associated compounds occurred (e.g., acetylpyrrol, 2-(5H)-furanone, pyrrolidinone, and trimethylpyrazine). Further, ethyl dodecanoate, heptanal, hexanoic acid, 4-methyl-2-heptanone, 2-pentanol acetate, 2-pentanone, 2-phenyl ethanol, and phenyl ethyl acetate were found in the chocolate-making samples. A PCA on the quantitative data of the chocolate-making samples explained 61.29% of the total variance (Figure 10A). The shell clusters of the NC cocoa beans differed from the ones of the SCI CFPs (Figure 10B). Upon further processing, the chocolate-making samples did not cluster substantially different from each other, whereas they did when compared to the cluster of the cocoa liquors of the AFSC VI CFPs. The effect of the different starter culture mixtures used was mainly visualized through the clusters of the cocoa liquors. Less distinctive clusters could be found for the subsequent steps of the chocolate-making process. The final chocolates did not reveal any distinctive clusters.

### 3.5. Correlations between microorganisms and metabolites identified

Although a correlation matrix has to be interpreted with caution, the links between the microorganisms and metabolites identified throughout the CFPs performed indicated correlations that were more clear for the bacteria than for the yeasts involved (Supplementary Figure S8), likely reflecting the wider yeast species diversity than the bacterial one. For instance, *Pichia* may be positively correlated with glycerol and dedicated VOCs, such as isoamyl acetate and phenylacetaldehyde (Supplementary Figure S8A). Concerning the bacterial communities, the most relevant correlations were found for *Liml. fermentum* (Supplementary Figure S8B). Indeed, glucose, fructose, citric acid, and malic acid correlated negatively with *Liml. fermentum*, reflecting their consumption, whereas positive correlations were found with 2,3-butanediol, D-lactate, L-lactate, and mannitol, reflecting their production. Alternatively, *A. pasteurianus* correlated positively with malic acid, citric acid, and gluconic acid, and negatively with lactic acid, mannitol, and 2,3-butanediol. However, those and other positive and negative correlations could be indirect effects, given the association of certain yeast and bacterial species.

## 4. Discussion

The current study dealt with the microbial diversity (cocoa pulp-bean mass) and VOC and non-VOC dynamics (cocoa pulps, cocoa beans during fermentation, and drying cocoa beans) of a spontaneous (NC) and five SCI CFPs, differing in the participating yeasts, carried out in duplicate with Trinitario cocoa in plastic vessels in Costa Rica. Besides a well-characterized *S. cerevisiae* strain, IMDO 050523 (Lefeber et al., 2012; Moens et al., 2014; Díaz-Muñoz et al., 2022), strains of *H. opuntiae* (IMDO 020003) and *P. kudriavzevii* (IMDO 060005) were involved, which were different from those used previously, namely *H. opuntiae* IMDO 040108 (Díaz-Muñoz et al., 2023) and *P. kudriavzevii* IMDO 020508 (Díaz-Muñoz et al., 2021). Based on the total cocoa pulp-bean mass DNA, a suitable high-resolution ASV approach, taking into account the full-length 16S rRNA gene (bacteria) and the ITS1 region of the fungal rRNA transcribed unit (yeasts), allowed to monitor the starter culture strains added along the fermentation courses. Furthermore, the chocolate-making allowed to determine the impact of the starter cultures used on the non-VOC and VOC profiles along cured cocoa bean processing.

Although biological duplicates did not differ significantly for all CFPs performed, the NC (F1 and F2) fermentation processes displayed the greatest variability, likely due to their spontaneous nature (Camu et al., 2007, 2008). Although the temporal succession of yeasts, LAB, and AAB, which is key for CFPs, is exposed to numerous environmental factors affecting the microbial species diversity and activities, the cocoa variety and post-harvest practices may be of influence too (Schwan and Wheals, 2004; Rottiers et al., 2019; De Vuyst and Leroy, 2020; Díaz-Muñoz and De Vuyst, 2022). The microbial community dynamics influence the pH and temperature courses and vice versa, as exemplified in the NC fermentation processes of the present study. In particular, whereas the increase of the temperature is mainly determined by the extent of AAB growth and will determine the survival of yeasts, the pH course depends on the LAB species diversity and metabolism, as, for instance, the consumption of citric acid in the beginning of CFPs results into a slightly higher pH and the heterofermentation of glucose leads to lactic acid and acetic acid, in turn decreasing the pH. Moreover, the predominance of *Liml. fermentum* IMDO 0611222 in all SCI CFPs had a substantial impact on the pH and temperature courses.

The spontaneous CFPs were particularly characterized by the growth of mainly *Weissella*, LAB that are naturally present in cocoa pulp-bean mass but do not prevail in general (Camu et al., 2007; Papalexandratou et al., 2019). As *Weissella* is not capable to reduce fructose to mannitol, due to the absence of the enzyme mannitol dehydrogenase (Gänzle, 2015), in contrast to *Liml. fermentum* (Verce et al., 2020), mannitol concentrations are then low and, hence, also acetic acid concentrations. Although *A. ghanensis* is known to be a fast oxidizer of ethanol to acetic acid, which is however twice as slow as *A. pasteurianus* (Moens et al., 2014), the F2 fermentation process did not show low acetic acid concentrations. Compared to the SCI CFPs, additional LAB species appeared in the NC fermentation processes besides *W. ghanensis*, namely *Leuc. pseudomesenteroides*, *Lc. lactis*, and *Lacp. plantarum*. Except for *Lc. lactis*, these LAB species often belong to the background LAB of fermenting cocoa pulp-bean mass (Camu et al., 2007; Papalexandratou et al., 2011a,b,c, 2013; Moreira et al., 2013; Díaz-Muñoz et al., 2021, 2023). Their presence is the result of environmental contamination, as they can be associated with the cocoa pods, plantain leaves, and even the farmers’ hands and machetes used, all providing an inoculation source for the cocoa pulp-bean mass, whether or not further prevailing during spontaneous CFPs (Camu et al., 2007, 2008; Papalexandratou et al., 2011a,b,c, 2013; Maura et al., 2016; Visintin et al., 2016; Lee et al., 2019; Díaz-Muñoz et al., 2021, 2023). Although *Leuconostoc* spp. may decrease the proportion of lactobacilli at the beginning of a fermentation course (Corsetti and Settanni, 2007; Jung et al., 2012), they do not survive long-term acidification (Corsetti et al., 2007; Van der Meulen et al., 2007). Yet, their citrate consumption, which is also the case for *Weissella* (Lefeber et al., 2011b), may explain their (transient) occurrence during CFPs, *in casu* the NC and PC fermentation processes (Lefeber et al., 2011a; Ouattara et al., 2017; Díaz-Muñoz et al., 2023). Whereas *Lacp. plantarum* often occurs in spontaneous CFPs (Papalexandratou et al., 2011a,b,c, 2013; Díaz-Muñoz et al., 2023), it does not perform well as a starter culture strain (Lefeber et al., 2010; Moreira et al., 2017). *Lactococcus lactis* seldomly occurs in spontaneous CFPs (Ostovar and Keeney, 1973).

Keeping the heat inside the fermenting cocoa pulp-bean mass is necessary, as a high temperature is – together with the diffusion of ethanol and in particular acetic acid into the cocoa beans – needed to kill the seed embryo. This was made possible during the vessel CFPs by coverage of the fermenting cocoa pulp-bean mass with plantain leaves, which resulted in an average fermentation temperature well above 40°C (except for the AFSC V fermentation processes) compared to the average maximal fermenting cocoa pulp-bean mass temperature of 37°C for all vessel CFPs carried out without banana leaves cover before (Díaz-Muñoz et al., 2021). Also the flow of sweatings from the vessels, which was promoted in the current study by regular mixing of the cocoa pulp-bean mass and an increased number of openings in the bottom of the vessels, determines the growth and activities of the residing microorganisms, in particular the LAB and AAB. A continuous flow of sweatings makes the fermenting matrix more aerobic, which, in combination with increased lactic acid concentrations produced by LAB, improves the growth of AAB (Camu et al., 2007, 2008; Papalexandratou et al., 2011a,b,c; Díaz-Muñoz et al., 2021). Again, AFSC V with its lower cocoa pulp-bean mass temperature compared to the other CFPs was an exception, which favoured the growth of yeasts and LAB and delayed the growth of AAB, implying no further pH increase after 24 h of fermentation due to the accumulation of lactic acid that was not overoxidized.

Through starter culture addition, more uniform presumptive counts were obtained for the yeast, LAB, and AAB communities and the microbial communities of the SCI CFPs were restricted to a few microbial species. The most abundant yeast genera of the SCI CFPs were *Saccharomyces*, *Hanseniaspora* and *Pichia*, which were part of the starter culture mixtures applied. Concerning the bacterial communities, *Tatumella* and/or *Pantoea* species occurred during initial stages of the CFPs and *Liml. fermentum* and *A. pasteurianus* along most part of the fermentation courses. The early growth of enterobacteria may contribute to the pulp liquefaction through pectinase activity (Papalexandratou et al., 2011c, 2013, 2019; Illeghems et al., 2015b; De Vuyst and Leroy, 2020). The in-depth ASV analysis showed that *Liml. fermentum* and *A. pasteurianus* in the SCI CFPs were mainly represented by the strains IMDO 0611222 and IMDO 0506386, respectively, which proved the successful inoculation of these starter culture strains thanks to their fitness to overrule the background microbiota and their effectiveness to steer CFPs. This confirmed the success of previous on-farm CFPs carried out with strains of these species in Ghana, Ivory Coast, Malaysia, and Costa Rica (Lefeber et al., 2012; Díaz-Muñoz et al., 2021, 2023). Indeed, although the highly fragmented genome assembly of *Liml. fermentum* IMDO 0611222 did not allow the identification of all 16S rRNA copies present in its genome, five different 16S rRNA gene copies present in the genome of this strain could be recovered by aligning the ASVs corresponding with this species to the genome of the inoculated strain (Verce et al., 2020). Nevertheless, this ASV-based strain-level monitoring is not always achieved with the same resolution, as was the case for *A. pasteurianus* in the present CFPs. This could be ascribed to a lack of intraspecies resolution of its 16S rRNA gene. As already shown for microbial community profiling by denaturing gradient gel electrophoresis of PCR amplicons targeting the 16S rRNA gene (Díaz-Muñoz et al., 2021), the use of the full-length 16S rRNA genes to infer high-quality ASVs is suitable to monitor AAB at species level but too conserved to resolve intraspecies variability. Nevertheless, care needs to be taken at interpreting relative abundances based on amplicon-based sequences from rRNA genes, especially for eukaryotes. Indeed, the number of copies of rRNA genes (locating the ITS1 region) varies considerably between fungal genera and this can influence any downstream processing (Ganley and Kobayashi, 2007). Additionally, it is challenging to determine the real number of rRNA genes present in fungal genomes, as most of them are highly fragmented. Even with highly contiguous fungal genomes, assembled using third-generation sequencing technologies, it is possible that the number of rRNA genes is underestimated during the assembly, as is the case for the *S. cerevisiae* IMDO 050523 genome (Díaz-Muñoz et al., 2022). However, the approximate number can be estimated using the sequencing coverage information (Ganley and Kobayashi, 2007; C. Díaz-Muñoz, Q. Vanderauwera, L. De Vuyst, and S. Weckx, unpublished results). Finally, the polyploid, heterozygous nature of fungal genomes makes it difficult to completely haplotype-phase the regions surrounding rRNA genes, which would result in a mixed nucleotide sequence from different alleles. This could be a possible explanation for the fact that only one of the three *P. kudriavzevii* ASVs showed 100% identity to the inoculated strain. Similarly, the *S. cerevisiae* 14 ASV that was also present in the CFPs that were not inoculated with the *S. cerevisiae* IMDO 050523 starter culture strain indicated that this presence had to be taken with care due to the low numbers of *S. cerevisiae* ASVs retrieved from the samples under study.

The AFSC V fermentation processes were characterized by the prevalence of *Limosilactobacillus* until their end because of inferior pulp liquefaction, providing less aerobic conditions, due to the sole presence of *H. opuntiae* as yeast starter culture. The lack of pectinase activity by *Hanseniaspora* species, avoiding pectin degradation, has been reported before (Seixas et al., 2019; Steenwyck et al., 2019). Nonetheless, pulp degradation could still be mitigated through endogenous enzymes (Camu et al., 2007; De Vuyst and Leroy, 2020; Díaz-Muñoz et al., 2021) or enterobacterial species present at early stages of the fermentation process (Illeghems et al., 2015b). This probably resulted in both an accumulation of non-volatile lactic acid and longstanding anaerobic conditions, thereby preventing the growth of *A. pasteurianus* IMDO 0506386, as a well-balanced ratio of the microbial consortium members is necessary to allow good AAB growth (Adler et al., 2014). Further, the accumulation of lactic acid probably led to a lower internal pH of the AFSC V cocoa beans. Up to now, few studies dealt with CFPs restricted to growth of *H. opuntiae* solely (Arana-Sánchez et al., 2015; Visintin et al., 2016; Díaz-Muñoz et al., 2023). Moreover, *H. opuntiae* has been reported to correlate negatively with *A. pasteurianus* (Mota-Gutierrez et al., 2018). Furthermore, *Hanseniaspora* could easily be outperformed by *Saccharomyces*, as was the case in the AFSC VI fermentation processes, notwithstanding the similar initial cell densities. Indeed, it has been hypothesized that *Hanseniaspora* growth can be inhibited by *Saccharomyces* because of cell–cell contact (Pietrafesa et al., 2020). It is likely that *Hanseniaspora* species lack competitiveness toward *S. cerevisiae*, and perhaps *P. kudriavzevii*, in particular because of their low heat and ethanol tolerance and their slow nutrient uptake (Díaz-Muñoz et al., 2021) that explains their occurrence in the initial fermentation phases of CFPs (Daniel et al., 2009; Papalexandratou et al., 2013; Díaz-Muñoz et al., 2021). Also another strain of *H. opuntiae*, IMDO 040108, as the sole yeast member of a cocoa starter culture mixture, has led to underfermented Costa Rican cocoa beans (Díaz-Muñoz et al., 2023). Despite their low competitiveness, the lack of fructose 1,6-bisphosphatase, phosphoenolpyruvate carboxykinase, and isocitrate lyase could cause the inability of *Hanseniaspora* species to grow on alternative carbon sources, such as glycerol and ethanol (Casal et al., 2008; Seixas et al., 2019). Finally, *Pichia* seemed to outperform *Saccharomyces* in the AFSC VIII fermentation processes, perhaps because of the ability of *Pichia* strains to produce killer toxins (Branco et al., 2014). A prevalence of *P. kudriavzevii* instead of *S. cerevisiae* during fermentation of cocoa pulp-bean mass has been shown before (Papalexandratou et al., 2011c).

The fungal communities present in the fresh cocoa pulp-bean mass, on genus level encompassing – in decreasing order of relative abundance and based on ASVs – *Pichia, Saccharomyces*, *Hanseniaspora*, *Wickerhamomyces*, *Acremonium,* and *Ambrosiozyma* are expected to be inoculated from the close environment as their bacterial counterparts. For example, *Acremonium*, an endophytic fungus, has been isolated hitherto from healthy cocoa leaves, stems and pods, and often represents yet undescribed species because species of this genus represent many anamorphs (Evans et al., 2003; Rojas et al., 2017). Further, the yeast *Ambrosiozyma* can produce cocoa flavour compounds at a multifold level, among which fusel alcohols (e.g., 2-methyl-1-butanol), ethyl esters (e.g., ethyl isobutryate), and acetate esters (e.g., butyl acetate), in comparison to *Saccharomyces* yeasts (Gamero et al., 2016). Similarly, species of *Wickerhamomyces* can be interesting cocoa flavour producers (Koné et al., 2016).

Carbohydrate consumption happened within 48 h of fermentation in all SCI CFPs of the present study, in contrast to the 96 h necessary for the spontaneous CFPs. An accelerated glucose and fructose consumption particularly occurred in the presence of the *S. cerevisiae* starter culture strain. Indeed, the species *S. cerevisiae* is known to be a fast carbohydrate consumer and, consequently, a high ethanol producer (Schwan, 1998; Moreira et al., 2013; Batista et al., 2015; Maura et al., 2016). Ethanol is a key metabolite during cocoa pulp-bean mass fermentation, as it has to be cross-fed to the AAB to be oxidized to acetic acid, another key metabolite of CFPs. As *H. opuntiae* was not able to fulfil a complete alcoholic fermentation as the sole yeast starter culture, particularly due to a slow carbohydrate consumption (AFSC V fermentation processes), it leads to underfermentation, confirming a former study applying another strain of *H. opuntiae* as the sole yeast member of a starter culture mixture for a SCI CFP of Costa Rican cocoa (Díaz-Muñoz et al., 2023) and former studies on citrus wines (Hu et al., 2020). It probably further explains scarce attempts to use *H. opuntiae* in cocoa starter cultures (Batista et al., 2015; Ho et al., 2018). In contrast, *P. kudriavzevii* IMDO 060005 as the sole yeast starter culture as well as in combination with *S. cerevisiae* IMDO 050523 caused a fast carbohydrate consumption, in contrast with another strain of the same species, *P. kudriavzevii* IMDO 020508, applied as member of a cocoa starter culture mixture in previous Costa Rican CFPs (Díaz-Muñoz et al., 2021). Given the enhanced prevalence of *P. kudriavzevii* IMDO 060005, this strain may be a better candidate cocoa yeast starter culture than the other one. Finally, carbohydrate consumption could also be ascribed to the LAB starter culture strain, *Liml. fermentum* IMDO 0611222, given the increased lactic acid and mannitol concentrations in the SCI CFPs. Also, this strain was responsible for an efficient citric acid consumption thanks to its citrate lyase activity (Bekal et al., 1998; Camu et al., 2007; Lefeber et al., 2011a,b; De Vuyst and Weckx, 2016; Ouattara et al., 2017; Verce et al., 2021). Other organic acids, such as acetic acid and succinic acid, also originated from the citrate metabolism of LAB, although yeasts and *Pantoea* could be responsible for succinic acid production too (Illeghems et al., 2015b; Dzialo et al., 2017; Figueroa-Hernández et al., 2019; Verce et al., 2020). Whereas acetic acid diffused into the beans, thereby decreasing the internal bean pH, lactic acid was taken up to a lesser extent and citric acid barely or not at all. However, lactic acid favoured AAB growth, which implies an indirect effect on the internal bean pH (Camu et al., 2007; Papalexandratou et al., 2011c). Hence, the final pH increase of the fermenting cocoa pulp-bean mass was the result of diffusion of both acetic acid and lactic acid into the cocoa beans, volatilization because of the heat and mixing of the fermenting cocoa pulp-bean mass, and further oxidation through AAB growth (Schwan and Wheals, 2004; De Vuyst and Leroy, 2020). The consumption of malic acid could be assigned to malolactic fermentation (Díaz-Muñoz et al., 2021), which could be confirmed by the presence of the malic enzyme in *Liml. fermentum* (Verce et al., 2020). Alternatively, it could be converted via fumaric acid into succinic acid with fumarate hydratase and fumarate reductase (Verce et al., 2020). Indeed, the lack of fumaric acid and an increased concentration of succinic acid throughout the cocoa fermentation course suggested its complete conversion via this pathway in all CFPs. The increase in malic acid concentrations at the end of all CFPs, except for AFSC V, could be attributed to *A. pasteurianus*, which is capable to produce malic acid (Bartowsky and Henschke, 2008; Illeghems et al., 2013). Finally, the occurrence of gluconic acid in all CFPs may be ascribed to its production from glucose by *Tatumella* and *Pantoea* (Kageyama et al., 1992; Adachi et al., 2003), by *Gluconobacter* (Papalexandratou et al., 2011b), or by *A. pasteurianus* 0506386 (Illeghems et al., 2013), and was present at high relative abundances in all SCI CFPs, in particular at the end (Moens et al., 2014). As gluconic acid can be consumed by *S. cerevisiae* (Peinado et al., 2003), and given the absence of this yeast species in the starter culture mixture of the AFSC VII fermentation processes, it occurred in higher concentrations in those CFPs.

The amino acids detected and quantified in the cocoa beans of all CFPs were isoleucine, leucine, valine, alanine, phenylalanine, and tyrosine. However, no significant dynamics in the amino acid concentrations were revealed during fermentation, probably due to the suboptimal pH course taking place in the cocoa beans. These cocoa flavour precursors are the result of aspartic endoprotease activity, releasing hydrophobic oligopeptides and likely optimally active in the initial fermentation phase given its optimal pH of 3.5, followed by exocarboxypeptidase activity, liberating a multifold of hydrophilic peptides and hydrophobic amino acids, optimally taking place at a pH of 5.5–6.0, which was never reached in any of the CFPs (Biehl et al., 1993; Voigt et al., 1994, 2018; De Vuyst and Leroy, 2020).

Although enterobacteria, *in casu Tatumella* and *Pantoea*, are known to produce biogenic amines (Delgado-Ospina et al., 2020), the increase of the concentrations of the biogenic amines tryptamine and tyramine in the cocoa beans may be ascribed to the LAB metabolism in the cocoa pulp. This was reflected in their increasing trend with a decreasing cocoa pulp-bean mass pH. An increase of the concentrations of these biogenic amines in cocoa beans has been described before (Oracz and Nebesny, 2014; De Brito et al., 2017). The low tryptamine and tyramine concentrations in the cocoa beans of the AFSC VII and AFSC VIII fermentation processes may be associated with the ability of *S. cerevisiae* and *P. kudriavzevii* to degrade biogenic amines in the cocoa pulp (Delgado-Ospina et al., 2021).

Finally, the wide array of VOCs produced in the cocoa pulp-bean mass, either by the plant itself or through microbial biosynthesis (Serra-Bonvehí, 2005; Camu et al., 2007; Moreira et al., 2016; Qin et al., 2017; Utrilla-Vazquez et al., 2020), define the flavour of cured cocoa beans when their uptake through diffusion has taken place (Kadow et al., 2013; Chetschik et al., 2018; Castro-Alayo et al., 2019; De Vuyst and Leroy, 2020). The nature of these VOCs is particularly determined by the yeast activities that have taken place (Ho et al., 2014; Díaz-Muñoz et al., 2021, 2023). Indeed, less variation occurred when *H. opuntiae* IMDO 020003 was used as yeast starter culture, which confirmed previous findings with the *H. opuntiae* IMDO 040108 strain (Díaz-Muñoz et al., 2023). Yet, the latter strain has been associated with a dedicated production of the alcohols 2/3-methyl-1-butanol and 2,3-butanediol (Díaz-Muñoz et al., 2021). Whereas VOCs formed through microbial metabolism in the cocoa pulps (in particular higher alcohols and esters) can diffuse into the cocoa beans, migration of ethanol and acetic acid and increasing temperatures cause the liberation of endogenous enzymes that induce flavour precursor formation, which becomes more relevant in chocolate-making processes (Ho et al., 2014; De Vuyst and Leroy, 2020). However, when *H. opuntiae* IMDO 020003 was combined with *S. cerevisiae* IMDO 050523 (AFSC VI), more cocoa-related higher alcohols and esters (e.g., phenylethyl alcohol and isoamyl acetate, respectively) were produced. This could pinpoint the importance to always include a strain of *S. cerevisiae* in a starter culture mixture to ferment cocoa. Yet, studies on wine fermentation have reported an increased production of higher alcohols when strains of the latter yeast species were combined with other yeast species (Luan et al., 2018). Hence, regarding its effect on the flavour profile, the role of *H. opuntiae* should not be neglected, as the variability to produce VOCs among different *H. opuntiae* strains is high (Capece et al., 2005; Tristezza et al., 2016). Also, *H. opuntiae* has been reported to increase the aroma complexity in wine (Jayani et al., 2005; Whitener et al., 2017), as non-*Saccharomyces* yeasts often have a higher potential to produce enzymes (e.g., esterases) to impact flavour (da Silva et al., 2005; Maturano et al., 2012; Qin et al., 2017). Nonetheless, the genus *Pichia* is known to contribute significantly to the flavour richness of cocoa (Koné et al., 2016; De Vuyst and Leroy, 2020). The application of *P. kudriavzevii* IMDO 020508 has shown an increased contribution of higher aldehydes, higher alcohols, and esters in Costa Rican cocoa (Díaz-Muñoz et al., 2021). The less pronounced impact on beneficial cocoa-related VOCs by the *P. kudriavzevii* IMDO 060005 strain used in the present study could be explained by the strain-dependent flavour potential of this species (Pereira et al., 2017). Apart from the unquestionable importance of yeasts in producing VOCs, also LAB and AAB do contribute to the cocoa flavour (De Vuyst and Leroy, 2020; Díaz-Muñoz et al., 2021, 2023). For instance, benzaldehyde present in the cocoa pulp-bean mass of all CFPs of the present study was probably produced from the amino acid phenylalanine by LAB (Hamdouche et al., 2019; Verce et al., 2020). Further, higher alcohols are also products of the amino acid metabolism by LAB (Smid and Kleerebezem, 2017). Finally, AAB are correlated with acetic acid, acetoin, and even phenylethyl alcohol production (Illeghems et al., 2013; Moens et al., 2014; Mota-Gutierrez et al., 2018; De Vuyst and Leroy, 2020). Next, dedicated compounds associated to specific CFPs in the current study, as explained above for the cocoa pulps, were also revealed in the cocoa beans, which could be ascribed to migration processes, not only ethanol and acetic acid but also glycerol and mannitol as shown in the present study (De Vuyst and Leroy, 2020; Díaz-Muñoz et al., 2021). Moreover, the presence of certain VOCs in the cocoa shells indicates an on-going diffusion process. Finally, tetramethylpyrazine was formed during drying, likely from acetoin (Díaz-Muñoz et al., 2021).

During chocolate-making noteworthy compounds that occurred and/or increased in concentration were 2-phenyl ethanol and phenyl ethyl acetate, which are linked to Maillard reactions typically occurring during roasting (Ascrizzi et al., 2017). The concentration decrease (e.g., tetramethylpyrazine) or disappearance (e.g., benzaldehyde) of some compounds was an indication of conching (Bastos et al., 2019). Conching also resulted in the oxidation of alcohols to the concomitant aldehydes and consecutive organic acids (Clark et al., 2020). Further, volatile aldehydes (e.g., heptanal), furanones (e.g., 4-hydroxy-2,5-dimethyl-3-(*2H*)-furanone), pyrazines (e.g., trimethylpyrazine), pyrrols (e.g., 2-acetyl-1-pyrroline), and pyridines originated from Strecker degradations (Stadler et al., 2003; Frauendorfer and Schieberle, 2008; Aprotosoaie et al., 2016). Differences in VOC profiles as a result of the starter culture mixtures applied were most distinguishable among the cocoa liquors instead of the final chocolates, indicating a great effect of further cocoa liquor processing, among which the conching step and the addition of commercial cocoa butter.

## 5. Conclusion

An amplicon sequence variant (ASV) approach based on full-length 16S rRNA gene (bacteria) and ITS1 (yeasts) sequencing led to more accurate and reliable data concerning precise monitoring of the microbial strains used for starter culture inoculation of cocoa pulp-bean mass. However, starter culture addition led to an enhanced fermentation course if *S. cerevisiae* IMDO 050523 was present in the mixture inoculated, thereby producing desirable flavour compounds, such as higher alcohols and esters. *Hanseniaspora opuntiae* IMDO 020003 as the sole yeast strain in the starter culture mixture led to underfermentation and an inferior VOC profile, mainly due to its low competitiveness during fermentation of the cocoa pulp-bean mass. Whilst *P. kudriavzevii* may contribute to a richer VOC profile, the *P. kudriavzevii* IMDO 060005 strain tested in the present study did not, confirming strain dependency concerning VOC production of this yeast species. Even though differences in VOCs could be revealed in the cocoa liquors, no significant effect on the final chocolates could be obtained, mainly due to a great impact of cocoa liquor processing during chocolate-making. Hence, optimization of the starter culture mixture and cocoa liquor processing seem to be of pivotal importance.

## Data availability statement

The datasets presented in this study can be found in online repositories. The names of the repository/repositories and accession number(s) can be found at: https://www.ebi.ac.uk/ena/browser/view/PRJEB57747.

## Author contributions

DV and CD-M performed the field experiments, wet lab work, the statistical analyses, and drafted the manuscript. CD-M performed the culture-independent and bioinformatic analysis. DV performed the metabolite analyses. CH, SW, and LV contributed to the coordination of the field experiments. SW and LV designed and supervised the experimental set-up. DV, CD-M, SW, and LV revised the manuscript. DV and LV edited the manuscript. LV was responsible for funding. All authors read and approved the final version of the manuscript.

## Funding

This work was supported by the Research Council of the Vrije Universiteit Brussel (SRP7 and IOF3017 projects) and the Research Foundation Flanders (SBO project REVICO, S004617N). DV is the recipient of a PhD fellowship from the VUB.

## Conflict of interest

The authors declare that the research was conducted in the absence of any commercial or financial relationships that could be construed as a potential conflict of interest.

## Publisher’s note

All claims expressed in this article are solely those of the authors and do not necessarily represent those of their affiliated organizations, or those of the publisher, the editors and the reviewers. Any product that may be evaluated in this article, or claim that may be made by its manufacturer, is not guaranteed or endorsed by the publisher.

## Supplementary material

The Supplementary material for this article can be found online at: https://www.frontiersin.org/articles/10.3389/fmicb.2023.1232323/full#supplementary-material

## Footnotes

^1^ https://www.ebi.ac.uk/ena/browser/view/PRJEB57747
