# Supplementary material for: Yeast strains do have an impact on the production of cured cocoa beans, as assessed with Costa Rican Trinitario cocoa fermentation processes and chocolates thereof
Source: Front Microbiol. 2023 Aug 9;14:1232323. doi: 10.3389/fmicb.2023.1232323 (PMC10445768; doi:10.3389/fmicb.2023.1232323)
Supplement: Supplementary file 1 [file Data_Sheet_1.docx]

**Supplementary Material**

**Supplementary Table S1.** Local alignment of *Saccharomyces cerevisiae* amplicon sequence variants (ASVs) toward the genome sequence of *S. cerevisiae* IMDO 050523. The ASVs present at high relative abundances in the cocoa fermentation processes initiated with *S. cerevisiae* IMDO 050523 are shaded in grey.

| ASV | ASV  length (bp) | ITS1 copy A | | | ITS1 copy B | | |
| --- | --- | --- | --- | --- | --- | --- | --- |
|  |  | AL (bp) | Chromosome | ND | AL (bp) | Chromosome | ND |
| 1 | 382 | 382 | XII | 1 | 304 | XII | 1 |
| 2 | 382 | 382 | XII | 2 | 304 | XII | 1 |
| 3 | 382 | 382 | XII | 2 | 304 | XII | 1 |
| 4 | 382 | 382 | XII | 1 | 304 | XII | 1 |
| 5 | 382 | 382 | XII | 2 | 304 | XII | 1 |
| 6 | 382 | 378 | XII | 1 | 304 | XII | 1 |
| 7 | 382 | 382 | XII | 1 | 304 | XII | 1 |
| 8 | 382 | 381 | XII | 1 | 304 | XII | 1 |
| 9 | 365 | 365 | XII | 2 | 304 | XII | 2 |
| 10 | 365 | 365 | XII | 1 | 304 | XII | 1 |
| 11 | 366 | 366 | XII | 3 | 305 | XII | 3 |
| 12 | 272 | 272 | XII | 0 | 211 | XII | 1 |
| 13 | 365 | 365 | XII | 1 | 304 | XII | 2 |
| 14 | 365 | 365 | XII | 0 | 304 | XII | 1 |
| 15 | 383 | 385 | XII | 2 | 324 | XII | 3 |
| 16 | 382 | 374 | XII | 0 | 313 | XII | 1 |
| 17 | 366 | 366 | XII | 2 | 305 | XII | 2 |

AL, alignment length; ND, nucleotide length

**Supplementary Table S2.** Local alignment of *Pichia kudriavzevii* amplicon sequence variants (ASVs) toward the genome sequence of *P. kudriavzevii* IMDO 060005. The ASVs present at high relative abundances in the cocoa fermentation processes initiated with *P. kudriavzevii* IMDO 060005 are shaded in grey.

| ASV | ASV  length (bp) | ITS1 copy A | | | ITS1 copy B | | |
| --- | --- | --- | --- | --- | --- | --- | --- |
|  |  | AL (bp) | Chromosome | ND | AL (bp) | Chromosome | ND |
| 1 | 106 | 106 | III | 2 | 106 | I | 3 |
| 2 | 106 | 106 | III | 0 | 106 | I | 1 |
| 3 | 106 | 106 | III | 1 | 106 | I | 2 |
| 4 | 106 | 106 | III | 1 | 106 | I | 2 |
| 5 | 106 | 106 | III | 2 | 106 | I | 1 |
| 6 | 106 | 106 | III | 3 | 106 | I | 2 |
| 7 | 107 | 107 | III | 2 | 107 | I | 1 |
| 8 | 106 | 106 | III | 1 | 106 | I | 0 |
| 9 | 106 | 106 | III | 2 | 106 | I | 1 |
| 10 | 106 | 106 | III | 3 | 106 | I | 2 |

AL, alignment length; ND, nucleotide length

**Supplementary Table S3.** Local alignment of *Hanseniaspora* amplicon sequence variants (ASVs) toward the genome sequence of *H. opuntiae* IMDO 020003. The ASVs present at high relative abundances in the cocoa fermentation processes initiated with *H. opuntiae* IMDO 020003 are shaded in grey.

| ASV | ASV  length (bp) | ITS1 copy A | | | ITS1 copy B | | |
| --- | --- | --- | --- | --- | --- | --- | --- |
|  |  | AL (bp) | Chromosome | ND | AL (bp) | Chromosome | ND |
| 1 | 310 | 309 | IV | 2 | 309 | IV | 3 |
| 2 | 310 | 309 | IV | 1 | 309 | IV | 2 |
| 3 | 309 | 308 | IV | 1 | 308 | IV | 2 |
| 4 | 309 | 308 | IV | 2 | 308 | IV | 1 |
| 5 | 293 | 294 | IV | 8 | 294 | IV | 9 |
| 6 | 293 | 293 | IV | 6 | 293 | IV | 7 |
| 7 | 293 | 293 | IV | 4 | 293 | IV | 5 |
| 8 | 293 | 293 | IV | 5 | 293 | IV | 6 |
| 9 | 293 | 293 | IV | 4 | 293 | IV | 5 |
| 10 | 292 | 292 | IV | 6 | 292 | IV | 7 |
| 11 | 293 | 293 | IV | 2 | 293 | IV | 3 |
| 12 | 292 | 292 | IV | 1 | 292 | IV | 2 |
| 13 | 292 | 292 | IV | 0 | 292 | IV | 1 |
| 14 | 293 | 293 | IV | 3 | 293 | IV | 4 |
| 15 | 293 | 293 | IV | 2 | 293 | IV | 3 |
| 16 | 293 | 293 | IV | 2 | 293 | IV | 3 |
| 17 | 293 | 293 | IV | 1 | 293 | IV | 2 |
| 18 | 293 | 293 | IV | 2 | 293 | IV | 3 |
| 19 | 290 | 292 | IV | 8 | 292 | IV | 7 |
| 20 | 292 | 292 | IV | 1 | 292 | IV | 0 |

AL, alignment length; ND, nucleotide length

a

**Supplementary Table S4.** Concentrations of the non-volatile organic compounds in the chocolate making samples originating from 120-h cocoa fermentation processes. performed in vessels with Trinitario cocoa in Costa Rica, followed by eight days of drying. The type of fermentation process (F1-F12) is as explained in the legend of Figure 1.

| Fermentation process | Source | Non-volatile organic compounds | | | | | | |  | | |  | |  |  |
| --- | --- | --- | --- | --- | --- | --- | --- | --- | --- | --- | --- | --- | --- | --- | --- |
| Carbohydrates and sugar alcohols |  | Fructose  (mg/g) | Glucose  (mg/g) | Sucrose  (mg/g) | Glycerol  (mg/kg) | Mannitol  (mg/kg) | | *Myo*-inositol  (mg/kg) | | |  | | |  |  |
| Cocoa shells | NC (F1) | 1.4 | 1.0 | 0.7 | 1.8 | 0.5 | | 7.7 | | |  | | |  |  |
|  | NC (F2) | 1.4 | 1.0 | 0.7 | 1.8 | 0.5 | | 7.8 | | |  | | |  |  |
|  | PC (F3) | 5.1 | 0.9 | 1.8 | 5.5 | 12.0 | | 8.7 | | |  | | |  |  |
|  | PC (F4) | 2.9 | 0.2 | 0.7 | 1.4 | 9.8 | | 8.5 | | |  | | |  |  |
|  | AFSC VI (F7) | 3.9 | 1.5 | 4.5 | 5.5 | 10.8 | | 8.9 | | |  | | |  |  |
|  | AFSC VI (F8) | 3.1 | 1.0 | 3.2 | 6.6 | 13.0 | | 10.8 | | |  | | |  |  |
|  | AFSC VIII (F11) | 3.7 | 1.1 | 3.2 | 5.8 | 12.4 | | 9.3 | | |  | | |  |  |
|  | AFSC VIII (F12) | 4.9 | 2.1 | 6.1 | 7.0 | 13.1 | | 9.0 | | |  | | |  |  |
| Cocoa liquors | NC (F1) | 1.1 | 0.5 | 3.6 | 1.1 | 1.3 | | 2.6 | | |  | | |  |  |
|  | NC (F2) | 0.6 | 0.4 | 2.1 | 0.8 | 0.1 | | 2.8 | | |  | | |  |  |
|  | PC (F3) | 0.7 | 0.2 | 1.2 | 2.0 | 1.6 | | 2.5 | | |  | | |  |  |
|  | PC (F4) | 0.4 | 0.2 | 1.1 | 2.0 | 1.6 | | 2.7 | | |  | | |  |  |
|  | AFSC VI (F7) | 0.5 | 0.4 | 3.0 | 1.6 | 0.9 | | 2.9 | | |  | | |  |  |
|  | AFSC VI (F8) | 0.6 | 0.5 | 4.3 | 2.0 | 1.1 | | 2.9 | | |  | | |  |  |
|  | AFSC VIII (F11) | 0.5 | 0.3 | 2.0 | 2.0 | 1.3 | | 2.8 | | |  | | |  |  |
|  | AFSC VIII (F12) | 0.9 | 0.8 | 5.7 | 1.6 | 1.0 | | 3.4 | | |  | | |  |  |
| Conched cocoa liquors | NC (F1) | 1.3 | 0.6 | 4.2 | 1.1 | 1.3 | | 2.7 | | |  | | |  |  |
|  | NC (F2) | 0.5 | 0.3 | 1.8 | 0.7 | 0.1 | | 2.5 | | |  | | |  |  |
|  | PC (F3) | 0.8 | 0.2 | 1.2 | 2.0 | 1.6 | | 2.5 | | |  | | |  |  |
|  | PC (F4) | 0.5 | 0.3 | 1.5 | 2.0 | 1.6 | | 2.7 | | |  | | |  |  |
|  | AFSC VI (F7) | 0.5 | 0.5 | 3.2 | 1.3 | 0.8 | | 2.5 | | |  | | |  |  |
|  | AFSC VI (F8) | 0.3 | 0.3 | 1.6 | 1.4 | 0.8 | | 2.2 | | |  | | |  |  |
|  | AFSC VIII (F11) | 0.5 | 0.4 | 2.5 | 1.8 | 1.2 | | 2.6 | | |  | | |  |  |
|  | AFSC VIII (F12) | 0.8 | 0.7 | 5.6 | 1.6 | 1.0 | | 3.2 | | |  | | |  |  |
| Non-tempered chocolate | NC (F1) | 1.6 | 0.1 | 252.9 | 0.1 | 0.1 | | 0.5 | | |  | | |  |  |
|  | NC (F2) | 1.1 | 0.0 | 241.1 | 0.1 | 0.0 | | 0.4 | | |  | | |  |  |
|  | PC (F3) | 1.4 | 0.0 | 242.1 | 0.1 | 0.1 | | 0.5 | | |  | | |  |  |
|  | PC (F4) | 1.9 | 0.0 | 255.6 | 0.2 | 0.1 | | 0.6 | | |  | | |  |  |
|  | AFSC VI (F7) | 1.3 | 0.0 | 237.4 | 0.1 | 0.1 | | 0.4 | | |  | | |  |  |
|  | AFSC VI (F8) | 1.3 | 0.1 | 238.4 | 0.1 | 0.1 | | 0.4 | | |  | | |  |  |
|  | AFSC VIII (F11) | 1.5 | 0.0 | 243.3 | 0.2 | 0.1 | | 0.5 | | |  | | |  |  |
|  | AFSC VIII (F12) | 1.1 | 0.1 | 250.7 | 0.2 | 0.1 | | 0.4 | | |  | | |  |  |
| Final chocolate | NC (F1) | 25.6 | 30.6 | 329.1 | 0.1 | 0.1 | | 0.5 | | |  | | |  |  |
|  | NC (F2) | 24.1 | 28.7 | 340.5 | 1.0 | 0.0 | | 0.4 | | |  | | |  |  |
|  | PC (F3) | 17.0 | 20.6 | 269.7 | 0.2 | 0.1 | | 0.4 | | |  | | |  |  |
|  | PC (F4) | 25.8 | 30.7 | 339.9 | 0.2 | 0.1 | | 0.5 | | |  | | |  |  |
|  | AFSC VI (F7) | 28.7 | 34.2 | 349.4 | 0.1 | 0.1 | | 0.5 | | |  | | |  |  |
|  | AFSC VI (F8) | 19.0 | 23.3 | 300.6 | 0.2 | 0.1 | | 0.4 | | |  | | |  |  |
|  | AFSC VIII (F11) | 22.3 | 26.8 | 314.7 | 0.2 | 0.1 | | 0.4 | | |  | | |  |  |
|  | AFSC VIII (F12) | 21.0 | 25.7 | 320.1 | 0.2 | 0.1 | | 0.5 | | |  | | |  |  |
| Organic acids |  | Acetic acid  (mg/kg) | Citric acid  (mg/g) | Gluconic acid  (mg/g) | Glucuronic acid  (mg/g) | | Malic acid  (mg/g) | | | Oxalic  acid  (mg/g) | | | Succinic acid  (mg/g) | |  |
| Cocoa shells | NC (F1) | 4623.9 | 18.8 | 19.3 | 0.8 | 1.2 | | 9.2 | | | 2.0 | | |  |  |
|  | NC (F2) | 4124.8 | 17.2 | 19.0 | 1.0 | 1.2 | | 13.9 | | | 2.0 | | |  |  |
|  | PC (F3) | 4494.4 | 39.7 | 39.5 | 1.2 | 1.9 | | 10.2 | | | 3.2 | | |  |  |
|  | PC (F4) | 2843.1 | 19.4 | 24.2 | 0.9 | 1.2 | | 12.8 | | | 1.8 | | |  |  |
|  | AFSC VI (F7) | 3913.9 | 15.6 | 14.0 | 0.6 | 1.2 | | 8.2 | | | 2.2 | | |  |  |
|  | AFSC VI (F8) | 5324.9 | 33.8 | 30.6 | 1.1 | 2.4 | | 14.4 | | | 4.3 | | |  |  |
|  | AFSC VIII (F11) | 4279.4 | 17.6 | 18.7 | 0.7 | 1.1 | | 11.3 | | | 2.1 | | |  |  |
|  | AFSC VIII (F12) | 3761.2 | 22.9 | 5.6 | 0.4 | 1.8 | | 14.8 | | | 3.0 | | |  |  |
| Cocoa liquors | NC (F1) | 2874.4 | 14.1 | 1.4 | 0.0 | 0.8 | | 15.4 | | | 1.3 | | |  |  |
|  | NC (F2) | 2239.9 | 12.2 | 0.3 | 0.0 | 0.7 | | 17.5 | | | 1.1 | | |  |  |
|  | PC (F3) | 3030.4 | 10.7 | 1.0 | 0.1 | 0.8 | | 10.6 | | | 1.0 | | |  |  |
|  | PC (F4) | 3161.5 | 12.7 | 0.9 | 0.1 | 0.9 | | 16.0 | | | 1.3 | | |  |  |
|  | AFSC VI (F7) | 2323.2 | 13.1 | 0.0 | 0.1 | 1.0 | | 17.3 | | | 1.4 | | |  |  |
|  | AFSC VI (F8) | 3039.4 | 17.8 | 0.5 | 0.0 | 1.3 | | 22.4 | | | 2.0 | | |  |  |
|  | AFSC VIII (F11) | 2335.5 | 10.9 | 0.3 | 0.1 | 0.8 | | 16.3 | | | 1.2 | | |  |  |
|  | AFSC VIII (F12) | 2083.9 | 14.9 | 0.0 | 0.0 | 1.0 | | 18.7 | | | 1.6 | | |  |  |
| Conched cocoa liquors | NC (F1) | 2173.3 | 13.5 | 716.8 | 0.0 | 0.8 | | 16.0 | | | 1.3 | | |  |  |
|  | NC (F2) | 2497.4 | 19.9 | 0.5 | 0.1 | 1.0 | | 25.0 | | | 2.3 | | |  |  |
|  | PC (F3) | 2341.4 | 31.5 | 1.5 | 0.1 | 1.7 | | 33.3 | | | 2.8 | | |  |  |
|  | PC (F4) | 2457.8 | 12.1 | 0.4 | 0.1 | 0.9 | | 17.5 | | | 1.2 | | |  |  |
|  | AFSC VI (F7) | 2192.2 | 16.6 | 0.0 | 0.0 | 1.1 | | 23.0 | | | 1.6 | | |  |  |
|  | AFSC VI (F8) | 2561.9 | 12.2 | 0.1 | 0.1 | 0.9 | | 20.1 | | | 1.2 | | |  |  |
|  | AFSC VIII (F11) | 2592.9 | 11.7 | 0.1 | 0.0 | 0.7 | | 14.7 | | | 1.2 | | |  |  |
|  | AFSC VIII (F12) | 2083.2 | 20.9 | 0.0 | 0.0 | 1.4 | | 27.8 | | | 2.1 | | |  |  |
| Non-tempered chocolate | NC (F1) | 2929.2 | 13.6 | 366.0 | 0.0 | 0.7 | | 13.7 | | | 1.2 | | |  |  |
|  | NC (F2) | 1771.6 | 14.2 | 0.6 | 0.0 | 0.7 | | 15.8 | | | 1.5 | | |  |  |
|  | PC (F3) | 2686.3 | 20.7 | 1.5 | 0.0 | 1.1 | | 21.8 | | | 1.8 | | |  |  |
|  | PC (F4) | 3110.1 | 10.7 | 0.7 | 0.1 | 0.7 | | 14.7 | | | 0.9 | | |  |  |
|  | AFSC VI (F7) | 2138.9 | 14.6 | 0.3 | 0.1 | 0.9 | | 18.3 | | | 1.3 | | |  |  |
|  | AFSC VI (F8) | 2360.7 | 11.7 | 0.4 | 0.1 | 0.8 | | 15.3 | | | 1.2 | | |  |  |
|  | AFSC VIII (F11) | 2505.2 | 9.9 | 0.3 | 0.0 | 0.6 | | 13.0 | | | 1.0 | | |  |  |
|  | AFSC VIII (F12) | 1650.3 | 15.3 | 0.1 | 0.0 | 1.0 | | 19.0 | | | 1.5 | | |  |  |
| Final chocolate | NC (F1) | 1940.3 | 13.2 | 1.9 | 0.1 | 0.6 | | 10.9 | | | 1.1 | | |  |  |
|  | NC (F2) | 1830.8 | 8.0 | 0.7 | 0.0 | 0.4 | | 6.0 | | | 0.8 | | |  |  |
|  | PC (F3) | 2108.3 | 9.1 | 1.4 | 0.0 | 0.5 | | 9.5 | | | 0.8 | | |  |  |
|  | PC (F4) | 2010.3 | 8.9 | 0.9 | 0.0 | 0.5 | | 11.4 | | | 0.6 | | |  |  |
|  | AFSC VI (F7) | 1932.8 | 12.2 | 0.6 | 0.1 | 0.7 | | 13.0 | | | 1.0 | | |  |  |
|  | AFSC VI (F8) | 2073.0 | 10.9 | 0.7 | 0.0 | 0.6 | | 10.0 | | | 1.1 | | |  |  |
|  | AFSC VIII (F11) | 1999.0 | 7.9 | 0.5 | 0.0 | 0.4 | | 10.7 | | | 0.8 | | |  |  |
|  | AFSC VIII (F12) | 1684.9 | 9.1 | 0.4 | 0.0 | 0.5 | | 9.4 | | | 0.8 | | |  |  |


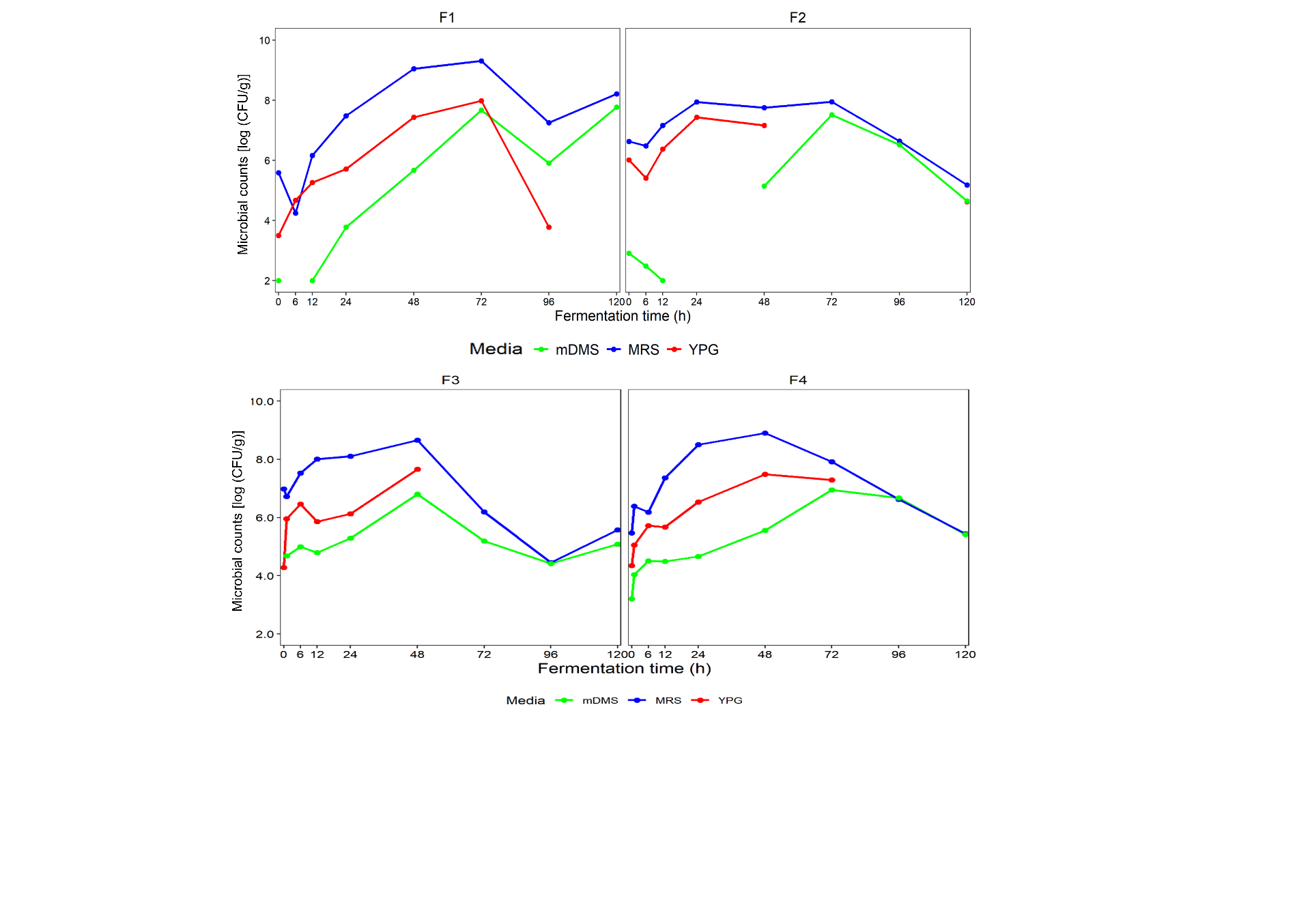


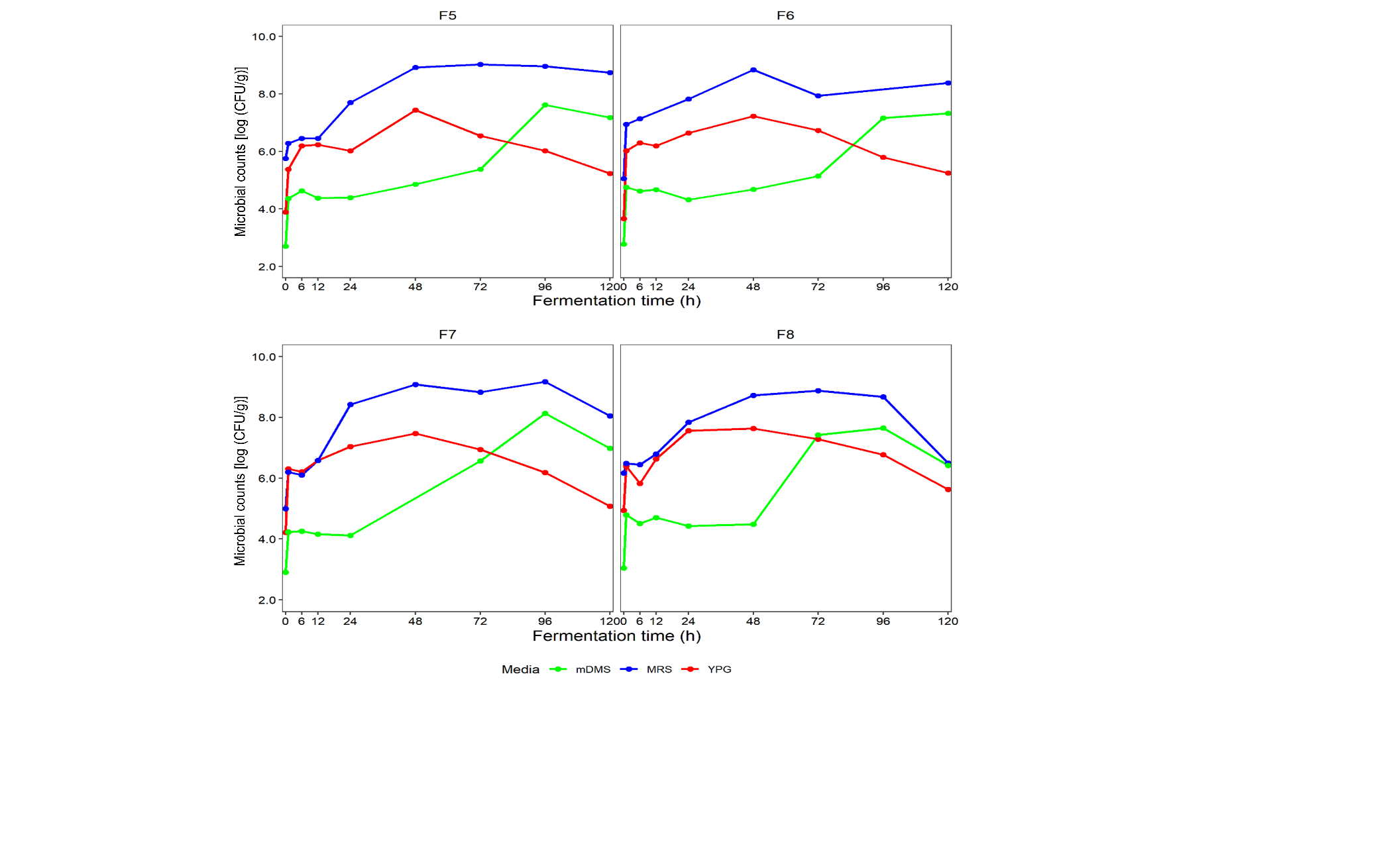


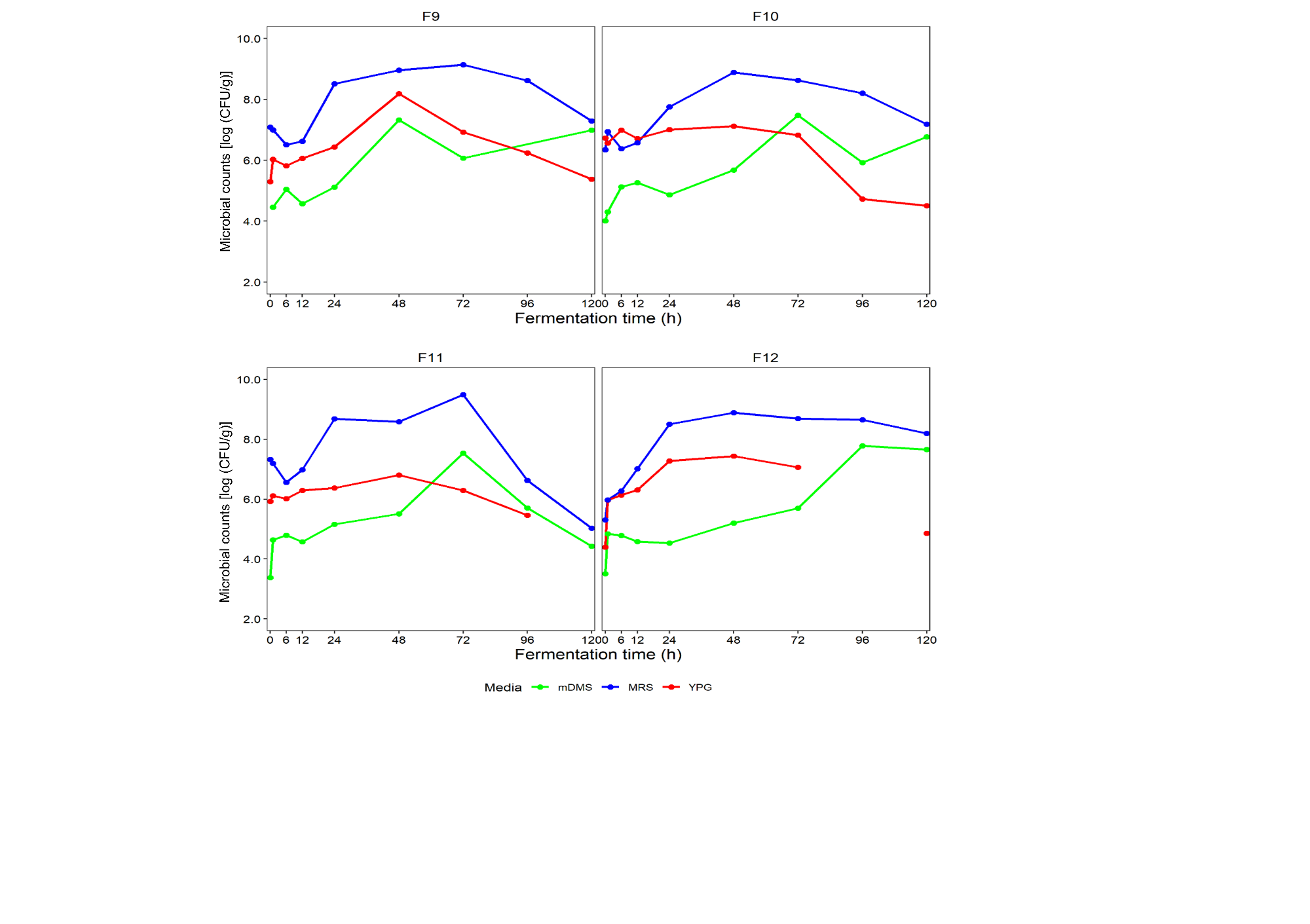


**Supplementary Figure S1.** Community dynamics of the presumptive yeasts, lactic acid bacteria (LAB), and acetic acid bacteria (AAB), expressed as colony-forming units (CFU/g) on yeast-peptone-glucose (YPG) agar medium, de Man-Rogosa-Sharpe (MRS) agar medium, and modified deoxycholate-mannitol-sorbitol (mDMS) agar medium, respectively, during 120-h cocoa fermentation processes, performed in vessels with Trinitario cocoa in Costa Rica. The type of fermentation process (F1-F12) is as explained in the legend of Figure 1.


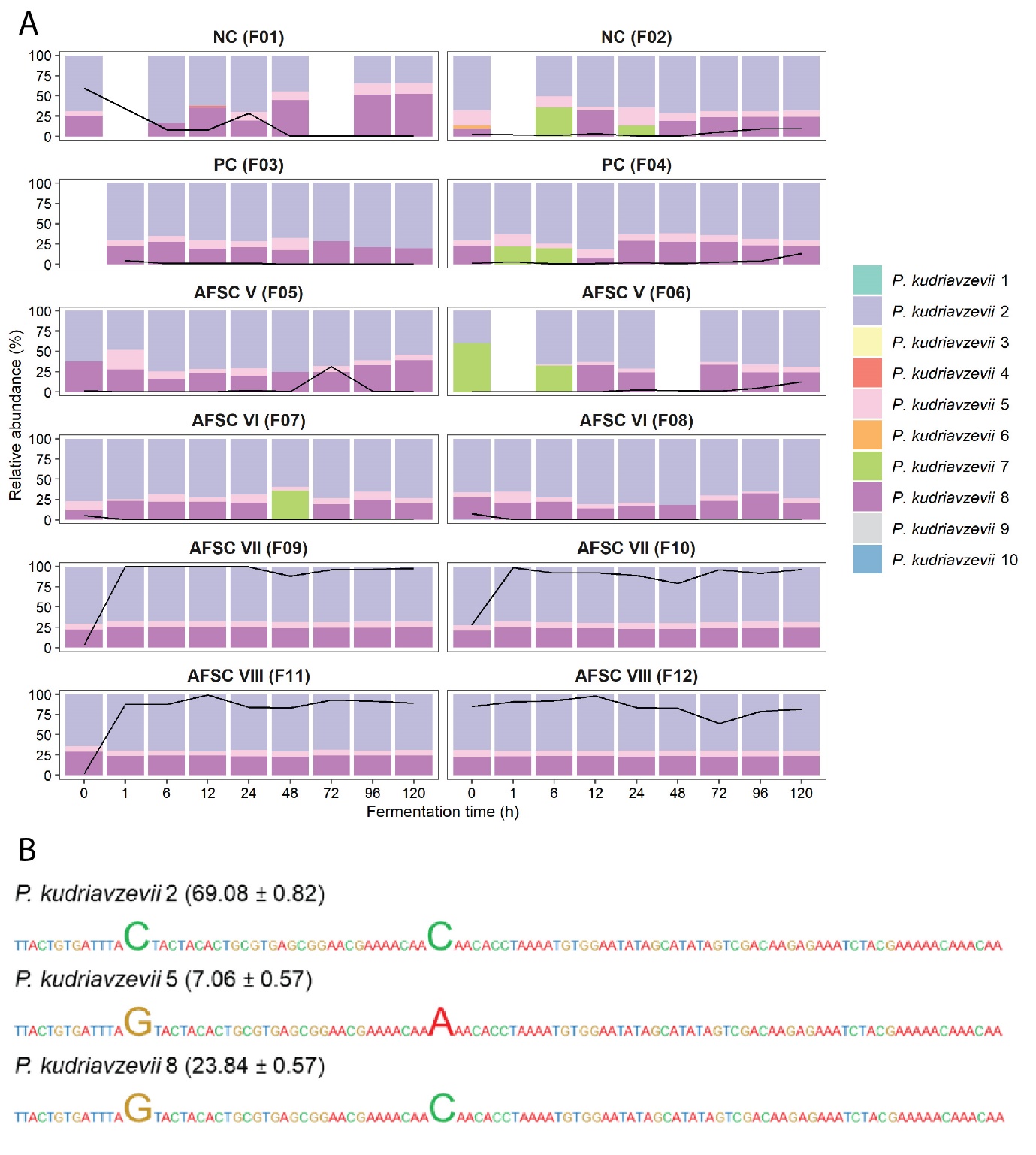


**Supplementary Figure S2.** Nucleotide sequences of the three amplicon sequence variants (ASVs) present in high relative abundances during the AFSC VII and AFSC VIII cocoa fermentation processes (CFPs), highlighting the single nucleotide variants (SNVs) found among them. The average percentage of relative abundance of each ASV during these CFPs is shown between brackets.


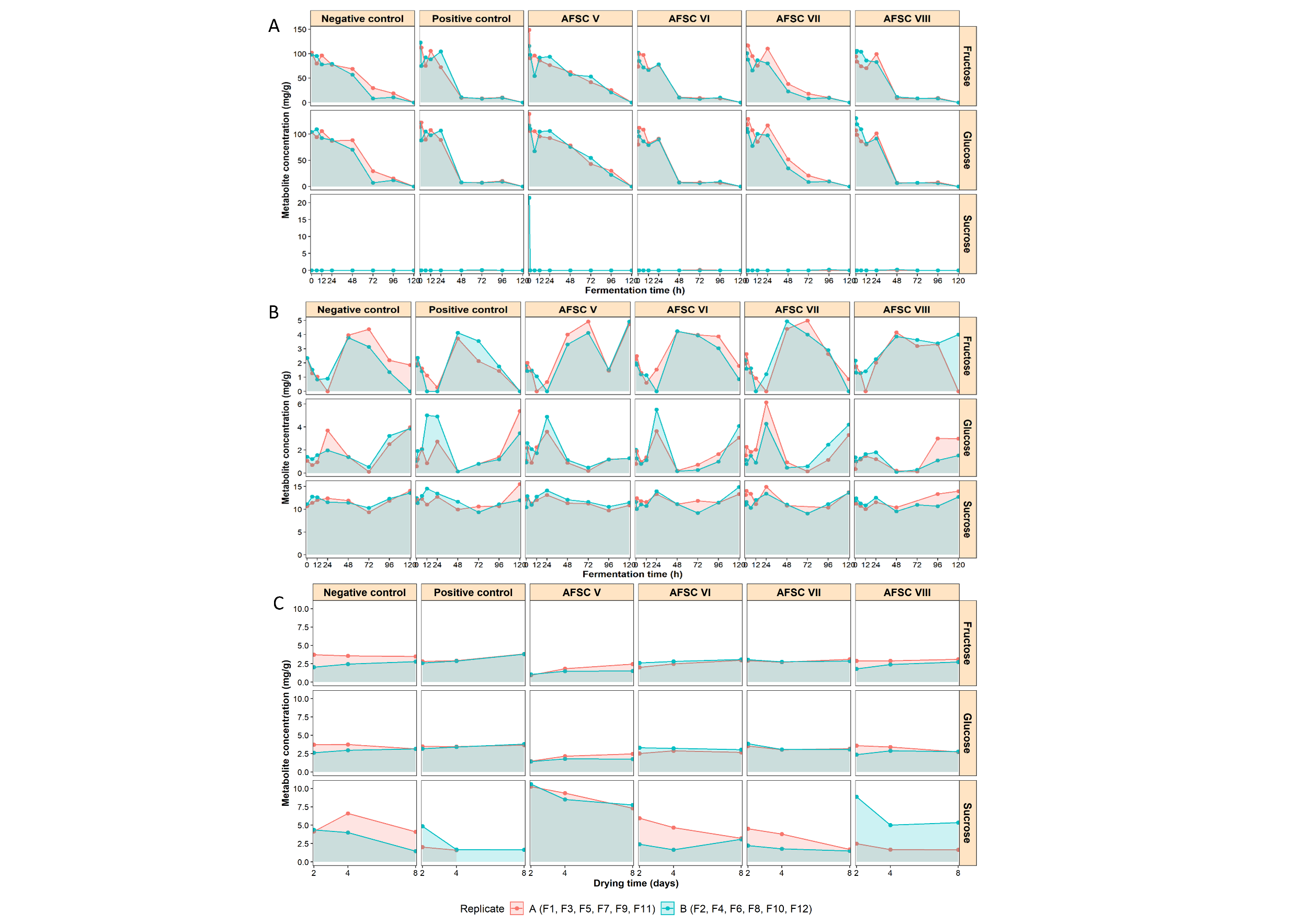


**Supplementary Figure S3.** Metabolite dynamics of carbohydrates (fructose, glucose, and sucrose) in the cocoa pulp **(A)** and beans **(B)** during 120-h cocoa fermentation processes, performed in vessels with Trinitario cocoa in Costa Rica, followed by eight days of drying (C). The type of fermentation process (F1-F12) is as explained in the legend of Figure 1.


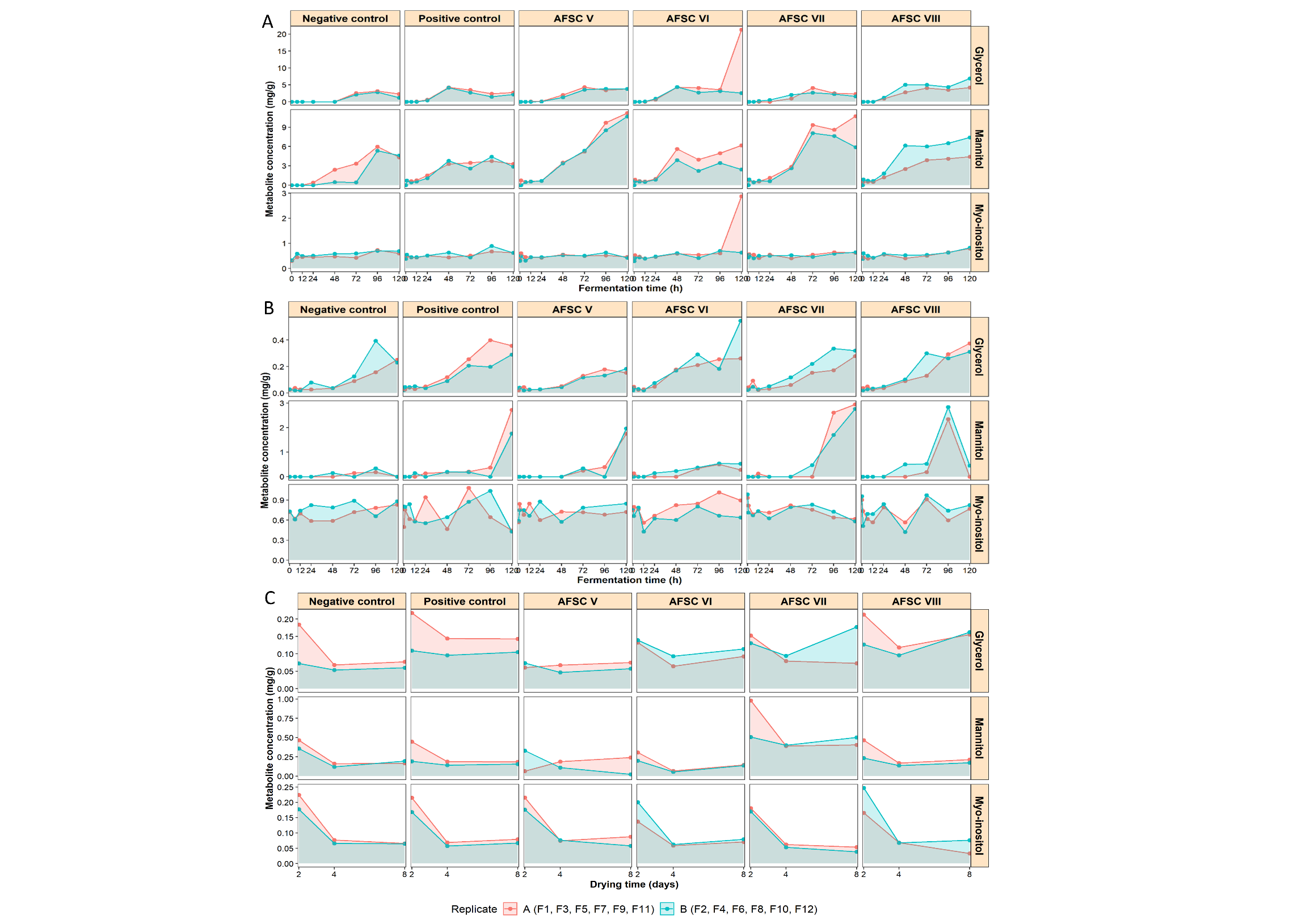


**Supplementary Figure S4.** Metabolite dynamics of sugar alcohols in the cocoa pulp **(A)** and beans **(B)** during 120-h cocoa fermentation processes, performed in vessels with Trinitario cocoa in Costa Rica, followed by eight days of drying **(C)**. The type of fermentation process (F1-F12) is as explained in the legend of Figure 1.


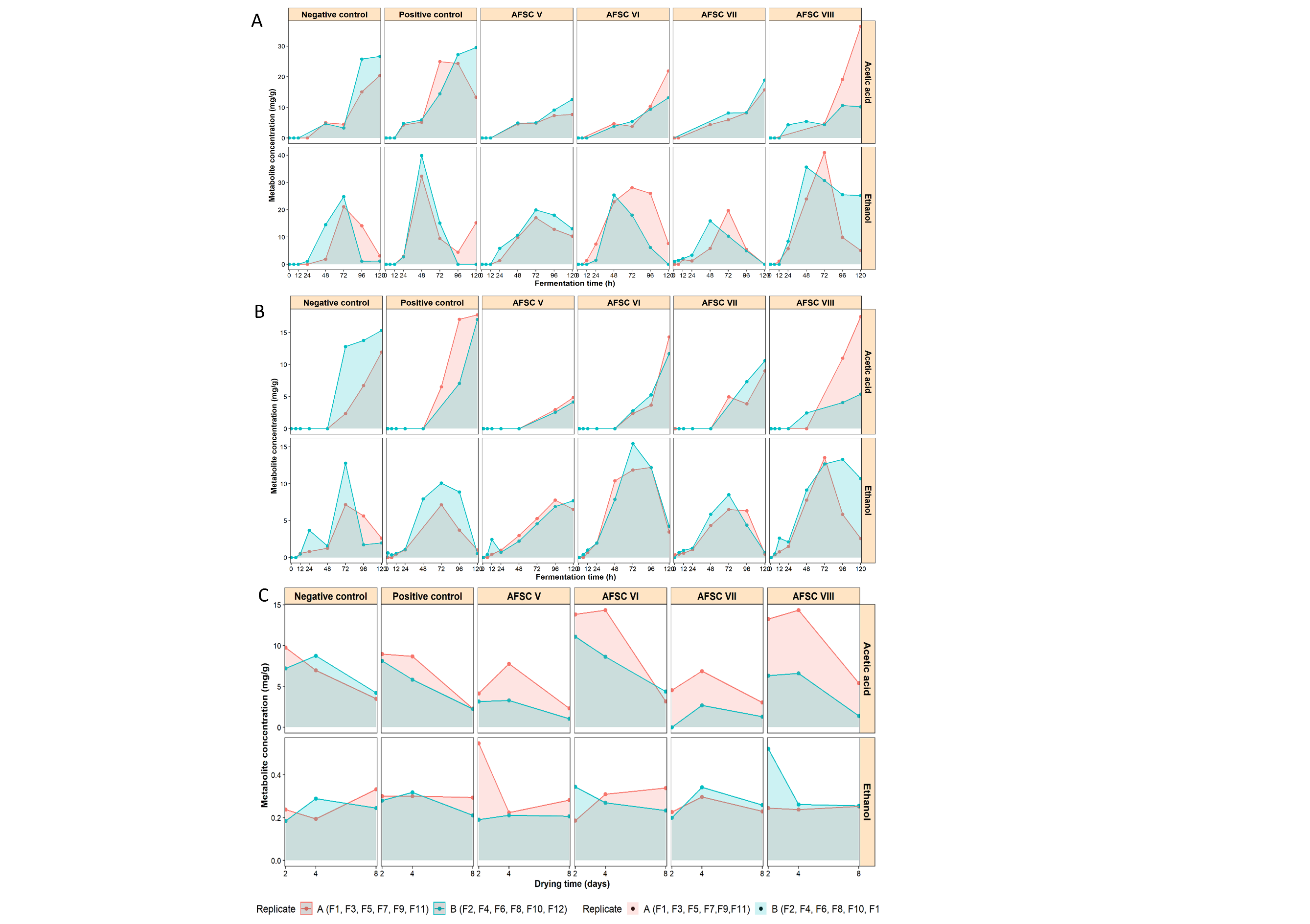


**Supplementary Figure S5.** Metabolite dynamics of short-chain fatty acids (acetic acid) and ethanol in the cocoa pulp **(A)** and beans **(B)** during 120-h cocoa fermentation processes, performed in vessels with Trinitario cocoa in Costa Rica, followed by eight days of drying **(C)**. The type of fermentation process (F1-F12) is as explained in the legend of Figure 1.


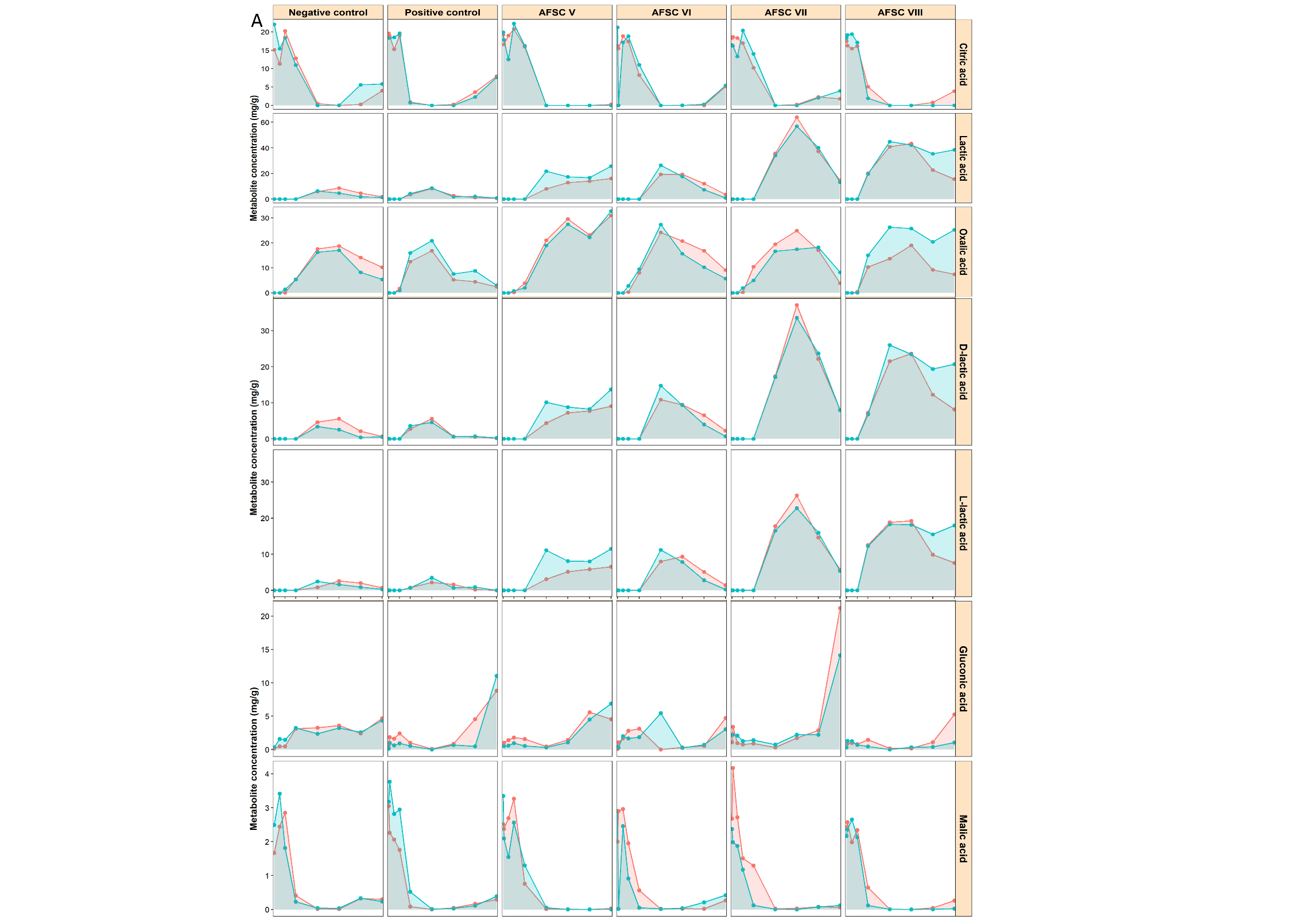


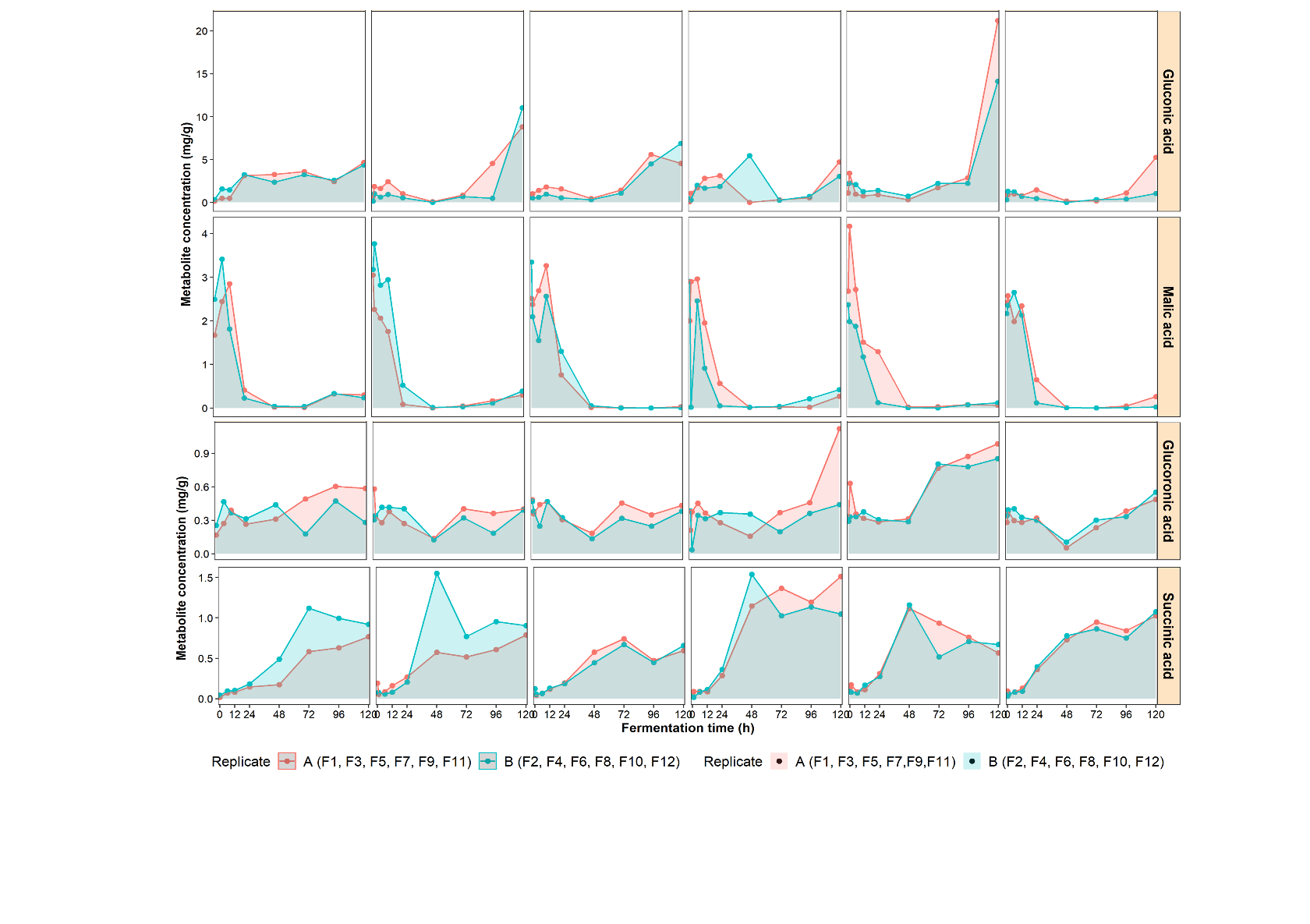


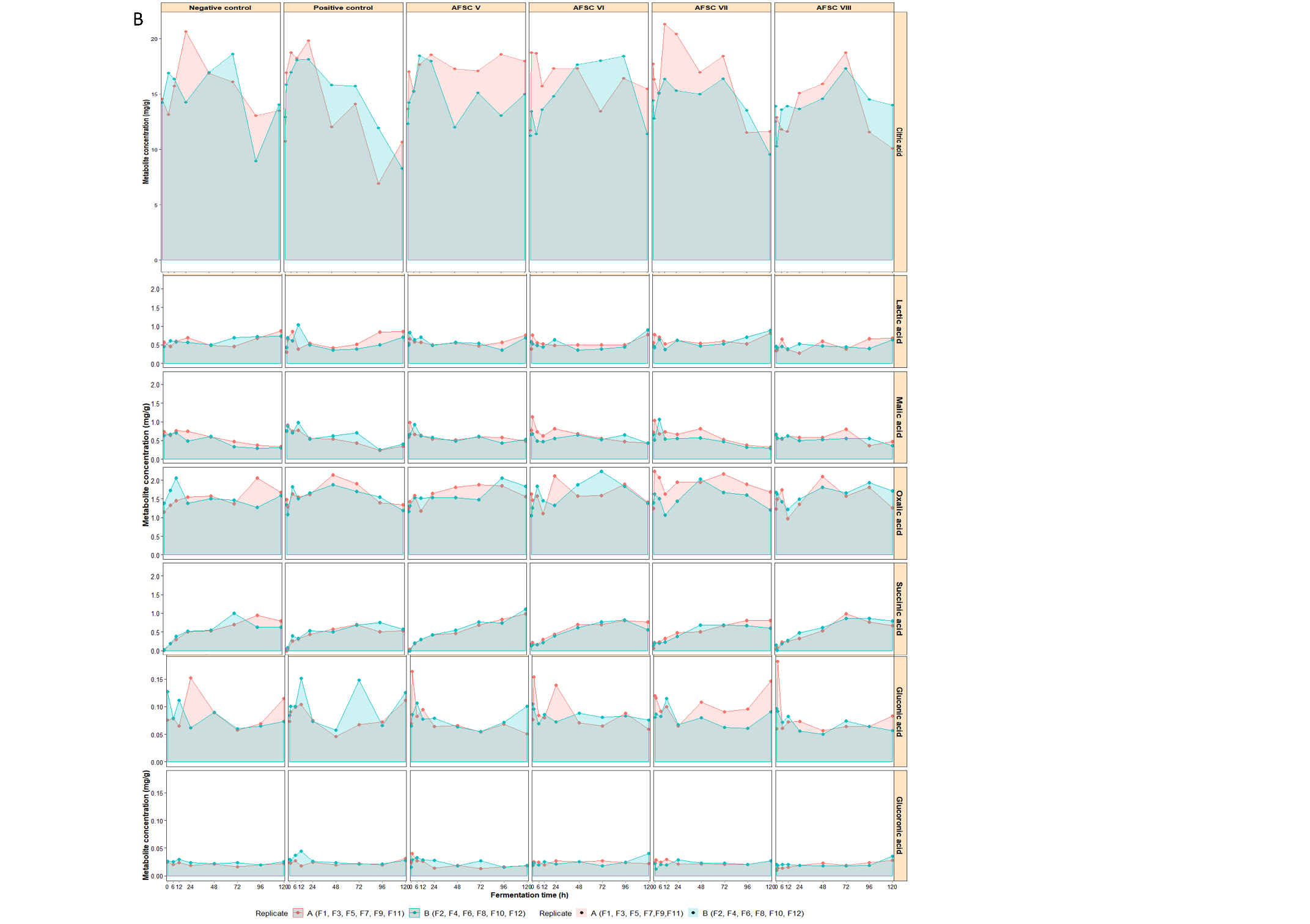


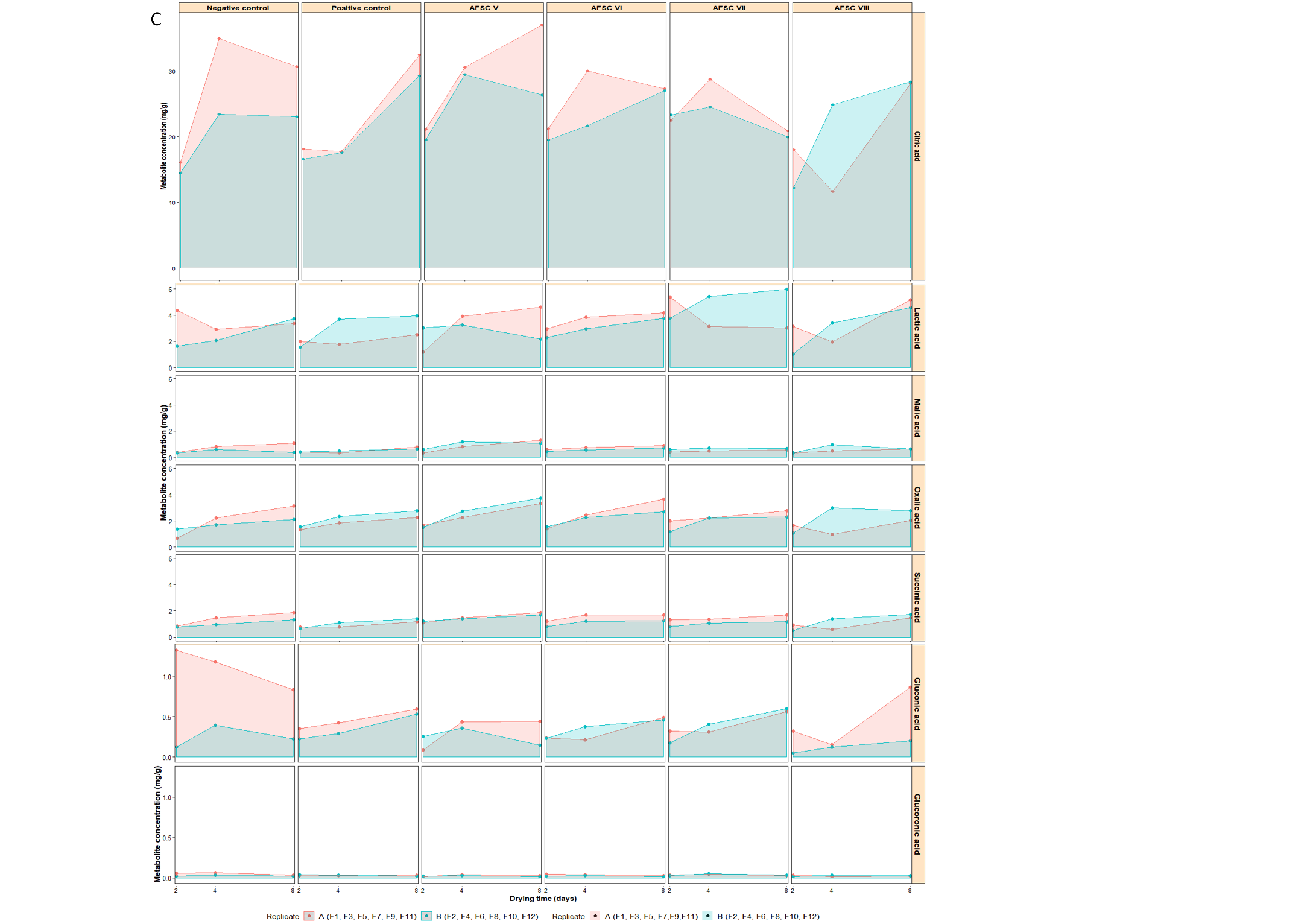
 **Supplementary Figure S6.** Metabolite dynamics of organic acids in the cocoa pulp **(A)** and beans **(B)** during 120-h cocoa fermentation processes, performed in vessels with Trinitario cocoa in Costa Rica, followed by eight days of drying **(C)**. The type of fermentation process (F1-F12) is as explained in the legend of Figure 1.


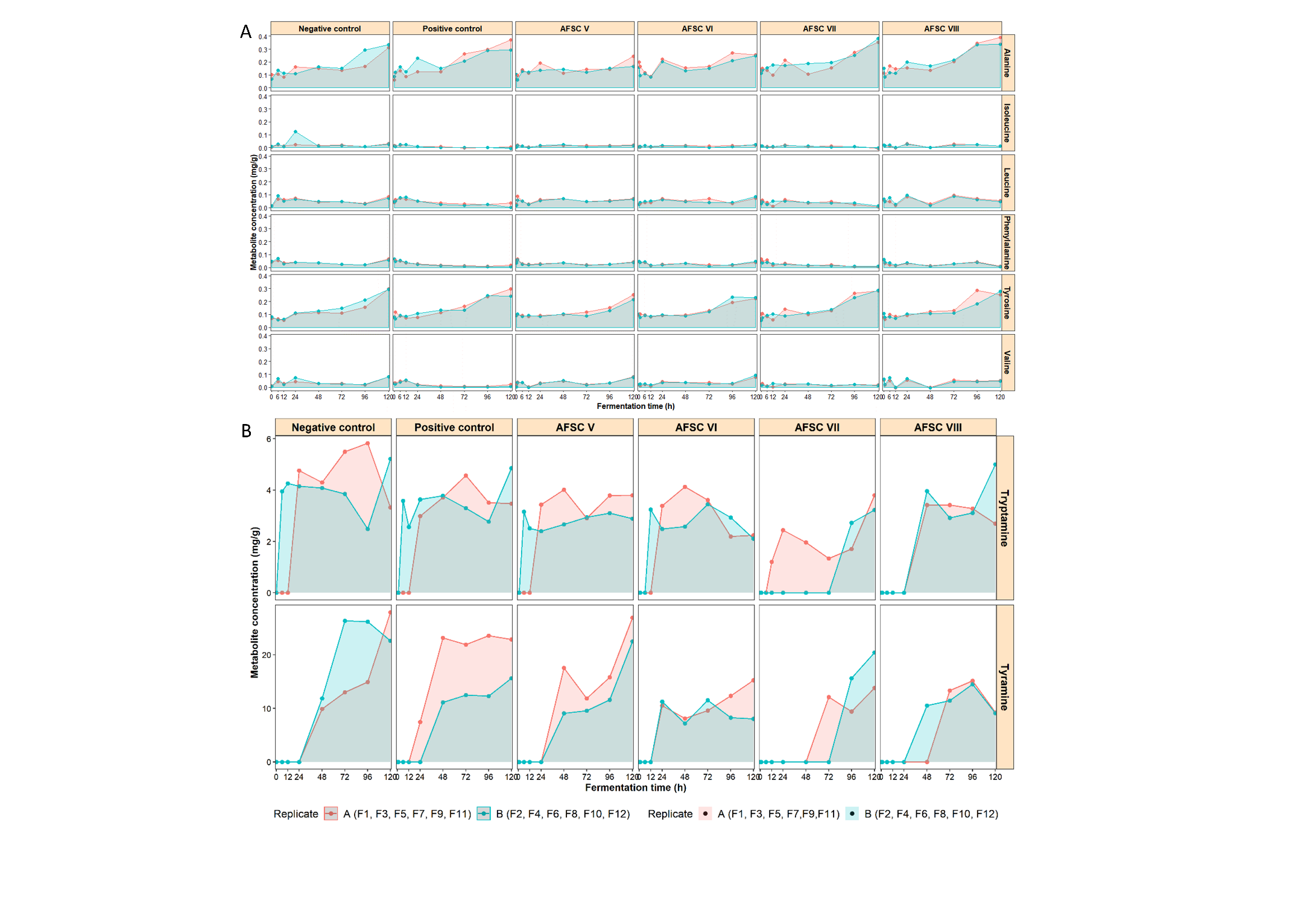


**B**

**Supplementary Figure S7**. Metabolite dynamics of amino acids **(A)** and biogenic amines **(B)** in cocoa beans during 120-h cocoa fermentation processes, performed in vessels with Trinitario cocoa in Costa Rica. The type of fermentation process (F1-F12) is as explained in the legend of Figure 1.


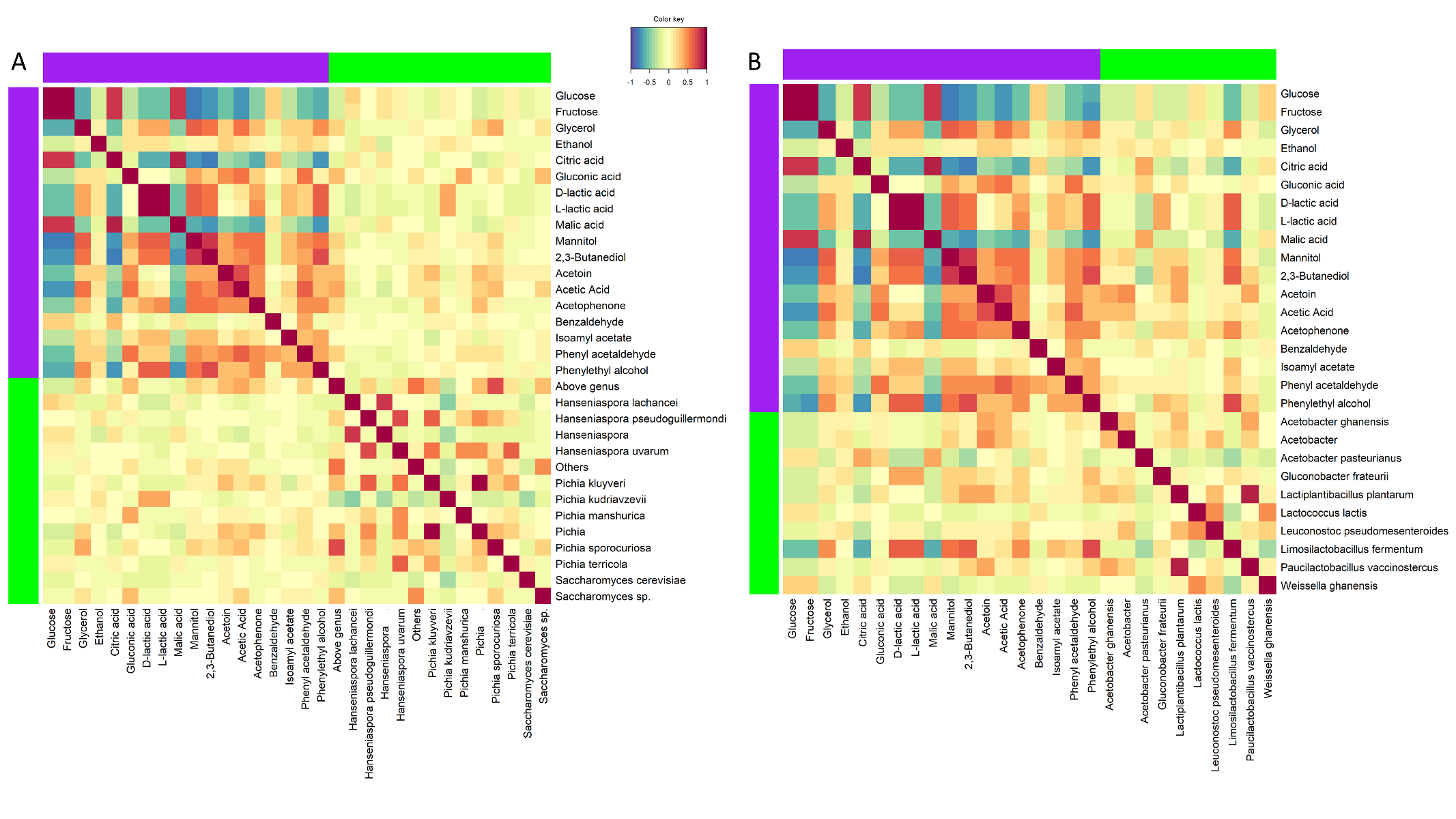


**Supplementary Figure S8.** Spearman correlation matrix between the microorganisms (green), namely yeasts **(A)** and bacteria **(B),** and the metabolites (violet) identified throughout the cocoa fermentation processes performed, as constructed with the mixOmics package (version 6.14.1; Rohart et al., 2017). Positive correlations are depicted in red whereas negative correlations are in blue; the color intensity represents the degree of correlation.

.
